# Ethyl­enedi­ammonium sodium tetra­kis­[bis­(ethyl­enedi­amine-κ^2^ *N*,*N*′)(oxalato-κ^2^ *O* ^1^,*O* ^2^)cobalt(III)] [penta­hydrogen di(phosphato­octa­deca­tungstate)] tetra­deca­hydrate

**DOI:** 10.1107/S160053681302583X

**Published:** 2013-10-16

**Authors:** Shuzhuo Zhang, Jing Wang, Yun Xu

**Affiliations:** a Department of Material Engineering Invention Examination, Patent Examination Cooperation Center of the Patent Office, SIPO, Beijing, People’s Republic of China

## Abstract

The title compound, Na(C_2_H_10_N_2_)[Co(C_2_O_4_)(C_2_H_8_N_2_)_2_]_4_[H_5_(P_2_W_18_O_62_)_2_]·14H_2_O, prepared under hydro­thermal conditions, consists of two Dawson-type [P_2_W_18_O_62_]^6−^ anions, four isolated [Co(en)_2_(ox)]^+^ cations (en = ethyl­enedi­amine and ox = oxalate), one Na^+^ cation, one [H_2_en]^2+^ cation, and a number of ordered (14) and disordered solvent water mol­ecules. The [P_2_W_18_O_62_]^6−^ polyoxidometalate anion has site symmetry 1 and contains two structurally distinct types of W atoms: *viz.* six W atoms on vertical pseudo-mirror planes grouped in two sets of three, and 12 equatorial W atoms that do not lie in the pseudo-mirror planes grouped in two sets of six. In each [Co(en)_2_(ox)]^+^ cation, the Co^III^ ion is coordinated by four N atoms from two en ligands and two O atoms from the ox ligands, completing a distorted octa­hedral structure. The sodium cation lies on an inversion centre and additionally links the complex cations and anions. In the crystal, the various units are linked by N—H⋯O and O—H⋯O hydrogen bonds, which together with C—H⋯O hydrogen bonds form a three-dimensional structure. The contribution of a region of disordered electron density, possibly highly disordered solvent water mol­ecules, to the scattering was removed with the SQUEEZE option of *PLATON* [Spek (2009 ▶). *Acta Cryst.* D**65**, 148–155]. To equilibrate the charges five H^+^ ions have been added to the polyoxidometalate. These H^+^ ions and the disordered solvent contribution were not included in the reported mol­ecular weight and density.

## Related literature

For general background to polyoxidometalate-based materials, see: Du *et al.* (2013 ▶); Dolbecq *et al.* (2010 ▶); Zheng & Yang (2012 ▶). For organic–inorganic hybrid materials constructed from components based on saturated polyoxidoanions and transition metal coordination complexes (TMCs), see: Liu *et al.* (2011 ▶); Wang *et al.* (2010 ▶). For related organic–inorganic hybrid compounds based on saturated Wells–Dawson-type polyoxidoanions and TMCs, and the synthesis of the POM precursor, Na_6_[P_2_W_18_O_62_]·19H_2_O, see: Wang *et al.* (2010 ▶); Contant (1990 ▶).
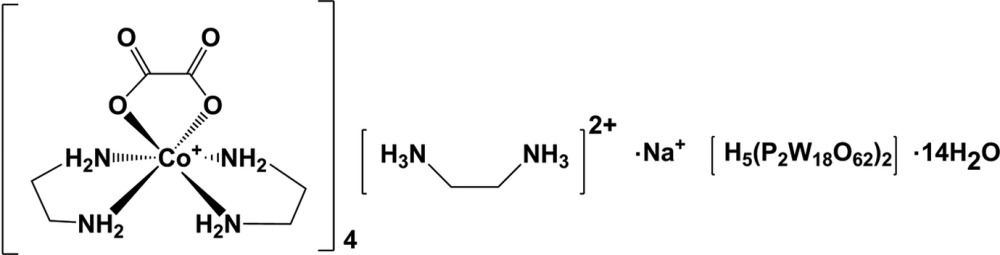


## Experimental

### 

#### Crystal data

Na(C_2_H_10_N_2_)[Co(C_2_O_4_)(C_2_H_8_N_2_)_2_]_4_[H_5_(P_2_W_18_O_62_)_2_]·14H_2_O
*M* *_r_* = 10132.09
Triclinic, 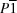

*a* = 14.5999 (4) Å
*b* = 16.5714 (4) Å
*c* = 18.0165 (4) Å
α = 83.693 (2)°
β = 84.586 (2)°
γ = 87.992 (2)°
*V* = 4311.88 (18) Å^3^
*Z* = 1
Mo *K*α radiation
μ = 24.42 mm^−1^
*T* = 293 K
0.12 × 0.06 × 0.05 mm

#### Data collection

Oxford Diffraction multiwire proportional diffractometer
Absorption correction: multi-scan (*CrysAlis RED*; Oxford Diffraction, 2009 ▶) *T*_min_ = 0.158, *T*_max_ = 0.375
49498 measured reflections
17862 independent reflections
12523 reflections with *I* > 2σ(*I*)
*R*_int_ = 0.057

#### Refinement

*R*[*F* ^2^ > 2σ(*F* ^2^)] = 0.040
*wR*(*F* ^2^) = 0.075
*S* = 0.96
17862 reflections
1096 parameters
12 restraints
H-atom parameters constrained
Δρ_max_ = 2.01 e Å^−3^
Δρ_min_ = −1.98 e Å^−3^

### 

Data collection: *CrysAlis CCD* (Oxford Diffraction, 2009 ▶); cell refinement: *CrysAlis CCD*; data reduction: *CrysAlis RED* (Oxford Diffraction, 2009 ▶); program(s) used to solve structure: *SHELXS97* (Sheldrick, 2008 ▶); program(s) used to refine structure: *SHELXL97* (Sheldrick, 2008 ▶); molecular graphics: *SHELXTL* (Sheldrick, 2008 ▶) and *DIAMOND* (Brandenburg, 2006 ▶); software used to prepare material for publication: *SHELXTL*.

## Supplementary Material

Crystal structure: contains datablock(s) 1, New_Global_Publ_Block. DOI: 10.1107/S160053681302583X/su2626sup1.cif

Additional supplementary materials:  crystallographic information; 3D view; checkCIF report

## Figures and Tables

**Table 1:** Hydrogen-bond geometry (Å, °)

*D*—H⋯*A*	*D*—H	H⋯*A*	*D*⋯*A*	*D*—H⋯*A*
N2—H2*A*⋯O100^i^	0.90	2.59	3.314 (14)	138
N2—H2*A*⋯O103^i^	0.90	2.07	2.940 (14)	163
N2—H2*B*⋯O38	0.90	2.26	3.115 (13)	157
O2*W*—H2*WA*⋯O57^ii^	0.85	2.44	2.919 (17)	117
N3—H3*C*⋯O103^i^	0.90	2.48	2.994 (14)	117
O2*W*—H2*WB*⋯O6*W*	0.85	2.04	2.86 (3)	162
N4—H4*D*⋯O1*W*	0.90	2.06	2.939 (18)	167
O3*W*—H3*WA*⋯O102^i^	0.85	2.16	3.000 (18)	172
O4*W*—H4*WA*⋯O12^iii^	0.85	2.14	2.983 (18)	173
N7—H7*D*⋯O5*W*	0.90	2.11	3.010 (17)	176
O4*W*—H4*WB*⋯O42	0.85	2.05	2.891 (16)	173
N5—H8*A*⋯O102	0.89	1.86	2.740 (15)	170
N5—H8*B*⋯O45^ii^	0.89	2.23	3.076 (13)	158
N5—H8*B*⋯O57^ii^	0.89	2.54	3.166 (14)	128
N5—H8*C*⋯O2*W*	0.89	2.05	2.881 (17)	155
N8—H8*E*⋯O7*W*	0.90	2.23	3.07 (2)	154
N9—H9*C*⋯O63^i^	0.90	2.12	3.023 (13)	175
N9—H9*D*⋯O49^ii^	0.90	2.20	3.088 (13)	170
C3—H3*A*⋯O29	0.97	2.49	3.417 (17)	160
C4—H4*B*⋯O10	0.97	2.47	3.396 (18)	160
C11—H11*A*⋯O33^ii^	0.97	2.52	3.376 (17)	147
C12—H12*A*⋯O25^ii^	0.97	2.51	3.297 (16)	139

Symmetry codes: (i) 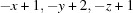; (ii) 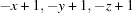; (iii) 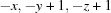.

**Fig. 1.**
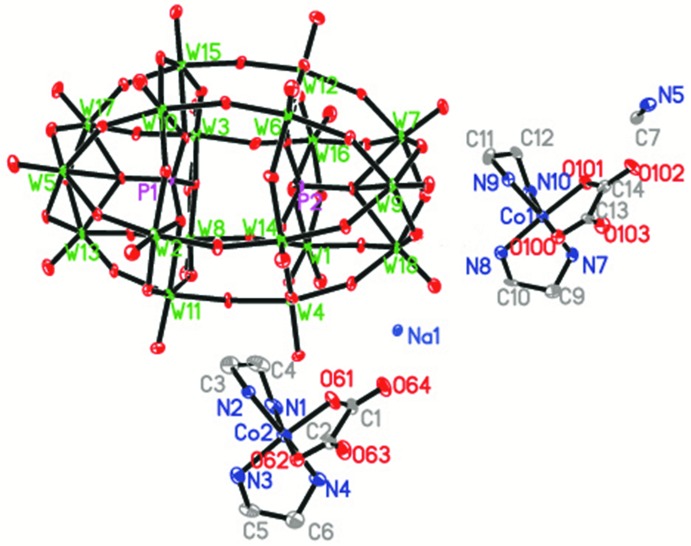
A view of the molecular structure of the asymmetric unit of the title compound, with partial atom labelling. Displacement ellipsoids are drawn at the 50% probability level. Only half of the diprotonated [H2en]2+ cation, situated about an inversion center, is shown (atoms N5 and C7). The Na+ ion is also situated on an inversion center. The H atoms and water molecules have been omitted for clarity.

**Fig. 2.**
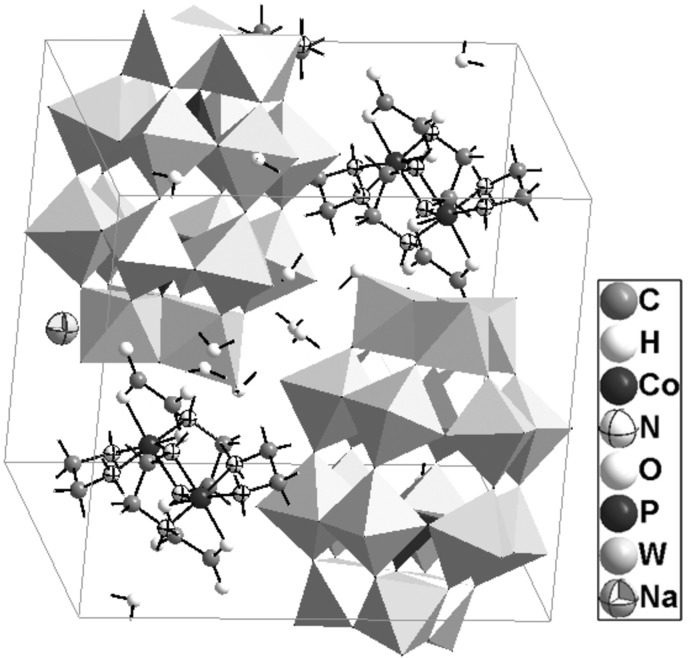
A view of the crystal packing of the title compound.

## supplementary crystallographic information

### 1. Comment

Polyoxidometalates (POMs), as one kind of significant metal oxide cluster have been attracting extensive interests in fields such as catalysis, medicine, biology, electrochemistry, electrochromism and magnetism for their enormous variety of properties. As their controllable size, shape, oxo-riched surfaces and the high-negative charges, POM's could be used as multi-linking sites or templates in constructing extended POM-based materials. Polyoxidotungstates (POT's) are the largest POM subclass, and to date, the two most studied heteropolyoxidometalates type have been the famous Keggin and Wells–Dawson families, which can be represented by [PW_12_O_40_]^3-^ and Wells-Dawson [P_2_W_18_O_62_]^6-^, respectively. In the past several decades, the synthetic combination of various POMs and transition metal cations or transition metal coordination complexes (TMCs) has been a powerful strategy and an intensive focus in POM chemistry, which leads to a rapidly growing class of functional organic–inorganic hybrid materials with a huge diversity of structures and realised applications in catalysis, photochemistry, and magnetochemistry. In this branch, a variety of organic–inorganic hybrid materials, constructed from components based on saturated Keggin polyoxidoanions and TMCs have been extensively reported. However, in contrast, the role of Wells–Dawson polyoxidoanions as the inorganic building units to construct such extended frameworks are much less common, the reason for which may be that the large size of these POT's decreases the electron density of the surface or the coordination ability of the surface oxygen atoms. In this paper, we report on the synthesis and crystal structure of the title organic–inorganic hybrid compound based on Wells–Dawson POMs and TMCs.

The asymmetric unit of the title compound, Fig. 1, consists of one [P_2_W_18_O_62_]^6-^ polyoxidoanion, two [Co(en)_2_(ox)]^+^ coordination cations, 0.5 Na^+^ cation, a half [H_2_en]^2+^ cation and seven lattice water molecules. The [P_2_W_18_O_62_]^6-^ cluster retains a classical α-Dawson-type structure, which may be described as two [α-PW_9_O_34_]^9-^ units, generated from the well known [α-PW_12_O_40_]^3-^ anion by removal of a set of three corner-sharing WO_6_ octahedra and fused into a cluster of virtual D_3h_ symmetry. The [P_2_W_18_O_62_]^6-^ polyoxidoanion contains only two structurally distinct types of W atoms: six polar W atoms on vertical mirror planes grouped in two sets of three, and twelve equatorial W atoms that do not lie on mirror planes grouped in two sets of six.

There are two crystallographically independent [Co(en)_2_(ox)]^+^ cations. The cobalt centers all exhibit a strongly distorted octahedral geometry, which is defined by four nitrogen atoms from two en ligands with Co–N bond lengths of 1.934 (11) to 1.961 (11) Å and two oxygen atoms from the oxalate ligands with Co–O bond lengths of 1.889 (9) to 1.927 (8) Å. The sodium cation lies on an inversion centre and links the complex cations and anions.

In the crystal, Fig. 2, the various units are linked by N-H···O and O-H···O hydrogen bonds, which together with C-H···O hydrogen bonds form a three-dimensional structure (Table 1).

### 2. Experimental

The saturated POM precursor, Na_6_[P_2_W_18_O_62_]^.^19H_2_O (I), was prepared according to the literature procedure (Wang *et al.*, 2010) and identified by IR spectra. A mixture of 0.406 g of (I), Co(Cl)_2_.6H_2_O (0.122 g), ethylenediamine (0.05 ml) and oxalic acid dehydrate (0.268 g) in H_2_O (10 ml) were stirred at room temperature for 60 min and transferred into a 30 ml Teflon-lined autoclave. It was heated under autogenous pressure at 373 K for two days. It was then allowed to cool to room temperature in air. Pink block-like crystals of the title compound, suitable for X-ray diffraction analysis, were obtained by filtration.

### 3. Refinement

All of the hydrogen atoms were placed in calculated positions and treated as riding atoms: O-H = 0.85 Å, N-H = 0.90 Å, C-H = 0.97 Å with Uiso(H) = 1.2Ueq(O/N/C). The contribution of highly disordered solvent water molecules [ca. 56 electrons for a volume of ca. 620 Å^3^] to the scattering was removed with the SQUEEZE routine of PLATON [Spek (2009). Acta Cryst. D65 148-155]. To equilibrate the charges five H^+^ ions have been added to the polyoxidometalate. These H^+^ ions and the disordered solvent contribution were not included in the reported molecular weight and density.

### Crystal data

**Table:**

Na(C_2_H_10_N_2_)[Co(C_2_O_4_)(C_2_H_8_N_2_)_2_]_4_·[H_5_(P_2_W_18_O_62_)_2_]·14H_2_O	*Z* = 1
*M**_r_* = 10132.09	*F*(000) = 4464
Triclinic, *P*1	*D*_x_ = 3.904 Mg m^−^^3^
Hall symbol: -P 1	Mo *K*α radiation, λ = 0.71073 Å
*a* = 14.5999 (4) Å	Cell parameters from 12336 reflections
*b* = 16.5714 (4) Å	θ = 2.8–26.5°
*c* = 18.0165 (4) Å	µ = 24.42 mm^−^^1^
α = 83.693 (2)°	*T* = 293 K
β = 84.586 (2)°	Block, pink
γ = 87.992 (2)°	0.12 × 0.06 × 0.05 mm
*V* = 4311.88 (18) Å^3^	

### Data collection

**Table:**

Multiwire proportional diffractometer	17862 independent reflections
Radiation source: fine-focus sealed tube	12523 reflections with *I* > 2σ(*I*)
Graphite monochromator	*R*_int_ = 0.057
phi and ω scans	θ_max_ = 26.5°, θ_min_ = 2.8°
Absorption correction: multi-scan (*CrysAlis RED*; Oxford Diffraction, 2009)	*h* = −18→16
*T*_min_ = 0.158, *T*_max_ = 0.375	*k* = −20→20
49498 measured reflections	*l* = −22→16

### Refinement

**Table:**

Refinement on *F*^2^	Primary atom site location: structure-invariant direct methods
Least-squares matrix: full	Secondary atom site location: difference Fourier map
*R*[*F*^2^ > 2σ(*F*^2^)] = 0.040	Hydrogen site location: inferred from neighbouring sites
*wR*(*F*^2^) = 0.075	H-atom parameters constrained
*S* = 0.96	*w* = 1/[σ^2^(*F*_o_^2^) + (0.0182*P*)^2^] where *P* = (*F*_o_^2^ + 2*F*_c_^2^)/3
17862 reflections	(Δ/σ)_max_ = 0.001
1096 parameters	Δρ_max_ = 2.01 e Å^−^^3^
12 restraints	Δρ_min_ = −1.98 e Å^−^^3^

### Special details

**Table:**

Geometry. Bond distances, angles etc. have been calculated using the rounded fractional coordinates. All su's are estimated from the variances of the (full) variance-covariance matrix. The cell esds are taken into account in the estimation of distances, angles and torsion angles
Refinement. Refinement of F^2^ against ALL reflections. The weighted R-factor wR and goodness of fit S are based on F^2^, conventional R-factors R are based on F, with F set to zero for negative F^2^. The threshold expression of F^2^ > 2sigma(F^2^) is used only for calculating R-factors(gt) etc. and is not relevant to the choice of reflections for refinement. R-factors based on F^2^ are statistically about twice as large as those based on F, and R- factors based on ALL data will be even larger.

### Fractional atomic coordinates and isotropic or equivalent isotropic displacement parameters (Å^2^)

**Table:**

	*x*	*y*	*z*	*U*_iso_*/*U*_eq_	
W1	0.33785 (3)	0.83307 (3)	0.43848 (3)	0.0187 (2)	
W2	0.15928 (3)	0.84213 (3)	0.08087 (3)	0.0201 (2)	
W3	0.11692 (3)	0.60561 (3)	0.43371 (3)	0.0202 (2)	
W4	0.36003 (3)	0.95497 (3)	0.25472 (3)	0.0186 (2)	
W5	−0.04200 (3)	0.70946 (3)	0.13226 (3)	0.0195 (2)	
W6	0.44450 (3)	0.64344 (3)	0.10820 (3)	0.0194 (2)	
W7	0.59839 (3)	0.65478 (3)	0.34232 (3)	0.0202 (2)	
W8	0.08595 (3)	0.80863 (3)	0.42223 (3)	0.0194 (2)	
W9	0.62006 (3)	0.76914 (3)	0.17495 (3)	0.0193 (2)	
W10	0.19271 (3)	0.61973 (3)	0.09293 (3)	0.0181 (2)	
W11	0.10783 (3)	0.93172 (3)	0.23866 (3)	0.0201 (2)	
W12	0.42288 (3)	0.53033 (3)	0.27617 (3)	0.0204 (2)	
W13	−0.09345 (3)	0.79987 (3)	0.29046 (3)	0.0202 (2)	
W14	0.41071 (3)	0.86512 (3)	0.09684 (3)	0.0196 (2)	
W15	0.17009 (3)	0.50775 (3)	0.26105 (3)	0.0193 (2)	
W16	0.36946 (3)	0.62933 (3)	0.44849 (3)	0.0208 (2)	
W17	−0.06329 (3)	0.59624 (3)	0.30233 (3)	0.0218 (2)	
W18	0.56863 (3)	0.85973 (3)	0.33407 (3)	0.0197 (2)	
P1	0.13154 (17)	0.71896 (17)	0.25456 (16)	0.0100 (8)	
P2	0.39783 (16)	0.74358 (17)	0.27104 (16)	0.0100 (8)	
O1	0.4970 (4)	0.6503 (4)	0.4159 (4)	0.020 (3)	
O2	0.4671 (4)	0.8420 (5)	0.4080 (4)	0.020 (3)	
O3	0.2108 (5)	0.8097 (5)	0.4439 (4)	0.022 (3)	
O4	0.2399 (5)	0.6271 (5)	0.4518 (4)	0.023 (3)	
O5	−0.0265 (4)	0.7945 (5)	0.3781 (4)	0.022 (3)	
O6	0.0050 (4)	0.6040 (5)	0.3848 (4)	0.019 (3)	
O7	0.0735 (5)	0.7023 (5)	0.4753 (4)	0.024 (3)	
O8	0.0403 (5)	0.8657 (5)	0.4906 (5)	0.032 (3)	
O9	0.0915 (5)	0.5351 (5)	0.5071 (5)	0.032 (3)	
O10	0.3349 (5)	0.8949 (5)	0.5087 (5)	0.028 (3)	
O11	0.3605 (5)	0.7301 (4)	0.4930 (4)	0.019 (3)	
O12	0.3868 (5)	0.5668 (5)	0.5257 (5)	0.035 (3)	
O13	0.6269 (5)	0.7620 (5)	0.3709 (4)	0.021 (3)	
O14	0.6653 (5)	0.6899 (5)	0.2474 (4)	0.020 (3)	
O15	0.6385 (5)	0.8506 (5)	0.2394 (5)	0.024 (3)	
O16	0.7121 (5)	0.7827 (5)	0.1083 (5)	0.027 (3)	
O17	0.5303 (4)	0.8425 (5)	0.1292 (4)	0.021 (3)	
O18	0.5555 (5)	0.6862 (5)	0.1351 (4)	0.022 (3)	
O19	0.4420 (5)	0.9089 (5)	0.0080 (5)	0.029 (3)	
O20	0.2809 (5)	0.8699 (5)	0.0917 (4)	0.022 (3)	
O21	0.3207 (4)	0.6144 (4)	0.1046 (4)	0.018 (3)	
O22	0.3997 (5)	0.9591 (4)	0.1504 (4)	0.019 (3)	
O23	0.1998 (4)	0.7337 (5)	0.0707 (4)	0.019 (3)	
O24	0.1123 (5)	0.9329 (4)	0.1318 (4)	0.021 (3)	
O25	0.1761 (5)	0.5172 (5)	0.1528 (5)	0.024 (3)	
O26	0.1821 (4)	0.6475 (5)	0.2200 (4)	0.019 (3)	
O27	0.1445 (4)	0.7173 (4)	0.3377 (4)	0.017 (3)	
O28	0.1592 (4)	0.8014 (5)	0.2115 (4)	0.019 (3)	
O29	0.1151 (5)	0.8872 (4)	0.3399 (4)	0.018 (3)	
O30	0.5362 (5)	0.5790 (5)	0.2962 (5)	0.022 (3)	
O31	0.3025 (5)	0.5131 (5)	0.2531 (4)	0.020 (3)	
O32	0.4577 (5)	0.4322 (5)	0.2960 (5)	0.030 (3)	
O33	0.4645 (5)	0.5451 (5)	0.1711 (5)	0.022 (3)	
O34	0.5031 (4)	0.7535 (4)	0.2779 (4)	0.018 (3)	
O35	0.4819 (4)	0.9274 (5)	0.2793 (4)	0.021 (3)	
O36	0.3651 (4)	0.8211 (4)	0.2237 (4)	0.018 (3)	
O37	0.3218 (5)	0.9085 (4)	0.3531 (4)	0.019 (3)	
O38	0.3600 (5)	1.0562 (5)	0.2637 (5)	0.027 (3)	
O39	0.0248 (4)	0.7096 (4)	0.2476 (4)	0.015 (2)	
O40	−0.1179 (4)	0.7807 (4)	0.1908 (4)	0.0154 (17)	
O41	−0.1326 (5)	0.6900 (5)	0.3254 (4)	0.022 (3)	
O42	−0.1373 (5)	0.5204 (5)	0.3329 (5)	0.030 (3)	
O43	−0.1055 (5)	0.7037 (5)	0.0574 (4)	0.026 (3)	
O44	0.0373 (5)	0.7964 (5)	0.0982 (4)	0.021 (3)	
O45	0.0604 (5)	0.6396 (4)	0.1072 (4)	0.021 (3)	
O46	0.2349 (4)	0.9498 (5)	0.2314 (4)	0.021 (3)	
O47	0.4073 (5)	0.7537 (5)	0.0827 (4)	0.023 (3)	
O48	0.3766 (5)	0.5602 (5)	0.3704 (5)	0.025 (3)	
O49	0.1638 (5)	0.4045 (5)	0.2787 (5)	0.025 (3)	
O50	0.3503 (4)	0.7355 (5)	0.3507 (4)	0.019 (3)	
O51	0.4875 (5)	0.6154 (5)	0.0227 (5)	0.030 (3)	
O52	0.1520 (5)	0.8824 (5)	−0.0097 (5)	0.032 (3)	
O53	0.6282 (5)	0.9312 (5)	0.3715 (5)	0.028 (3)	
O54	0.1942 (5)	0.5880 (5)	0.0055 (5)	0.029 (3)	
O55	−0.0105 (5)	0.8801 (5)	0.2480 (5)	0.025 (3)	
O56	−0.1881 (5)	0.8578 (5)	0.3164 (5)	0.032 (3)	
O57	−0.0918 (5)	0.6206 (5)	0.1999 (5)	0.027 (3)	
O58	0.0422 (5)	0.5345 (5)	0.2639 (5)	0.023 (3)	
O59	0.1687 (5)	0.5397 (4)	0.3586 (4)	0.021 (3)	
O60	0.0677 (5)	1.0281 (5)	0.2478 (5)	0.032 (3)	
O65	0.3883 (4)	0.6677 (4)	0.2326 (4)	0.018 (3)	
O66	0.6794 (5)	0.5958 (5)	0.3853 (5)	0.033 (3)	
Co2	0.25021 (11)	1.19551 (11)	0.42865 (10)	0.0289 (6)	
O61	0.3623 (5)	1.1326 (5)	0.4371 (5)	0.034 (3)	
O62	0.3081 (5)	1.2442 (5)	0.3373 (5)	0.029 (3)	
O63	0.4377 (5)	1.2326 (6)	0.2605 (5)	0.041 (3)	
O64	0.4987 (6)	1.1153 (6)	0.3728 (6)	0.047 (4)	
N1	0.1978 (7)	1.1349 (7)	0.5197 (6)	0.042 (4)	
N2	0.2013 (7)	1.1114 (6)	0.3764 (6)	0.032 (4)	
N3	0.1426 (7)	1.2683 (7)	0.4167 (6)	0.037 (4)	
N4	0.2958 (8)	1.2810 (6)	0.4816 (7)	0.041 (4)	
C1	0.4227 (8)	1.1489 (8)	0.3809 (8)	0.031 (4)	
C2	0.3885 (9)	1.2147 (8)	0.3195 (8)	0.032 (4)	
C3	0.1439 (9)	1.0549 (9)	0.4291 (9)	0.047 (6)	
C4	0.1781 (11)	1.0512 (10)	0.5048 (10)	0.061 (7)	
C5	0.1431 (10)	1.3356 (8)	0.4636 (8)	0.042 (5)	
C6	0.2422 (10)	1.3577 (9)	0.4623 (9)	0.051 (6)	
Co1	0.75551 (10)	0.74539 (10)	0.88977 (9)	0.0233 (5)	
O100	0.8081 (5)	0.8020 (5)	0.7976 (5)	0.029 (3)	
O101	0.8672 (5)	0.6799 (5)	0.8865 (5)	0.028 (3)	
O102	0.9952 (6)	0.6665 (6)	0.8108 (5)	0.044 (3)	
O103	0.9297 (6)	0.7946 (6)	0.7154 (5)	0.040 (3)	
N7	0.8064 (6)	0.8239 (6)	0.9455 (6)	0.031 (4)	
N8	0.6479 (7)	0.8172 (6)	0.8883 (6)	0.031 (4)	
N9	0.6956 (6)	0.6703 (6)	0.8350 (6)	0.032 (4)	
N10	0.7108 (7)	0.6771 (6)	0.9801 (6)	0.032 (4)	
C9	0.7515 (9)	0.8998 (8)	0.9410 (8)	0.043 (5)	
C10	0.6537 (8)	0.8759 (7)	0.9428 (7)	0.028 (4)	
C11	0.6425 (9)	0.6106 (9)	0.8874 (8)	0.040 (5)	
C12	0.6927 (8)	0.5962 (8)	0.9557 (8)	0.030 (4)	
C13	0.8866 (8)	0.7714 (7)	0.7746 (8)	0.027 (4)	
C14	0.9204 (9)	0.7003 (8)	0.8286 (7)	0.028 (4)	
N5	1.0313 (7)	0.5240 (7)	0.8966 (6)	0.039 (4)	
C7	0.9898 (9)	0.5365 (8)	0.9698 (8)	0.039 (5)	
O1W	0.4838 (9)	1.3078 (9)	0.4114 (8)	0.108 (7)	
O2W	1.2170 (9)	0.4589 (10)	0.8845 (9)	0.120 (8)	
O3W	−0.0429 (11)	1.3503 (10)	0.3525 (9)	0.135 (8)	
O4W	−0.3016 (9)	0.4337 (9)	0.3171 (9)	0.121 (8)	
O5W	1.0002 (9)	0.8663 (11)	0.8857 (9)	0.154 (9)	
O6W	1.3741 (14)	0.5597 (15)	0.8622 (19)	0.34 (2)	
O7W	0.4460 (13)	0.7681 (13)	0.9018 (11)	0.172 (11)	
Na1	0.50000	1.00000	0.50000	0.0182 (19)	
H1A	0.23750	1.13290	0.55520	0.0500*	
H1B	0.14540	1.15980	0.53660	0.0500*	
H2A	0.16720	1.13490	0.34040	0.0390*	
H2B	0.24820	1.08350	0.35410	0.0390*	
H3A	0.14740	1.00130	0.41200	0.0560*	
H3B	0.08010	1.07380	0.43130	0.0560*	
H3C	0.14240	1.28860	0.36830	0.0450*	
H3D	0.09100	1.24000	0.42950	0.0450*	
H4A	0.13190	1.02780	0.54260	0.0730*	
H4B	0.23350	1.01730	0.50670	0.0730*	
H4C	0.28890	1.26670	0.53140	0.0490*	
H4D	0.35600	1.28840	0.46770	0.0490*	
H5A	0.11790	1.31860	0.51450	0.0500*	
H5B	0.10680	1.38160	0.44350	0.0500*	
H6A	0.26430	1.38280	0.41290	0.0610*	
H6B	0.24900	1.39580	0.49850	0.0610*	
H7C	0.80730	0.80340	0.99380	0.0370*	
H7D	0.86480	0.83400	0.92680	0.0370*	
H8D	0.64510	0.84350	0.84210	0.0380*	
H8E	0.59670	0.78810	0.90000	0.0380*	
H9A	0.76960	0.93360	0.89490	0.0510*	
H9B	0.75990	0.92980	0.98300	0.0510*	
H9C	0.65760	0.69820	0.80430	0.0370*	
H9D	0.73840	0.64410	0.80660	0.0370*	
H10A	0.63230	0.85180	0.99260	0.0340*	
H10B	0.61510	0.92330	0.93020	0.0340*	
H10C	0.75330	0.67210	1.01350	0.0380*	
H10D	0.65880	0.69880	1.00140	0.0380*	
H11A	0.63820	0.56030	0.86500	0.0480*	
H11B	0.58080	0.63170	0.89970	0.0480*	
H12A	0.75030	0.56700	0.94490	0.0360*	
H12B	0.65600	0.56400	0.99520	0.0360*	
H7A	0.92380	0.54350	0.96810	0.0470*	
H7B	1.01280	0.58600	0.98470	0.0470*	
H8A	1.01880	0.56670	0.86430	0.0580*	
H8B	1.00900	0.47960	0.88210	0.0580*	
H8C	1.09200	0.51800	0.89780	0.0580*	
H1WA	0.51180	1.28040	0.44540	0.1300*	
H1WB	0.51570	1.34620	0.38810	0.1300*	
H2WA	1.22340	0.44320	0.84100	0.1430*	
H2WB	1.25860	0.49300	0.88670	0.1430*	
H3WA	−0.02720	1.34090	0.30760	0.1620*	
H3WB	−0.06990	1.39660	0.35150	0.1620*	
H4WA	−0.32980	0.43140	0.36070	0.1450*	
H4WB	−0.25190	0.45880	0.31760	0.1450*	
H5WA	1.03260	0.87490	0.92060	0.1850*	
H5WB	1.00100	0.90780	0.85330	0.1850*	
H6WC	1.40970	0.56620	0.82190	0.4130*	
H6WD	1.39750	0.52250	0.89150	0.4130*	
H7WA	0.44400	0.80850	0.92710	0.2070*	
H7WB	0.45670	0.72550	0.93080	0.2070*	

### Atomic displacement parameters (Å^2^)

**Table:**

	*U*^11^	*U*^22^	*U*^33^	*U*^12^	*U*^13^	*U*^23^
W1	0.0157 (2)	0.0251 (3)	0.0166 (3)	−0.0004 (2)	−0.0040 (2)	−0.0054 (2)
W2	0.0187 (2)	0.0238 (3)	0.0179 (3)	0.0017 (2)	−0.0061 (2)	−0.0003 (2)
W3	0.0195 (2)	0.0257 (3)	0.0152 (3)	−0.0048 (2)	−0.0014 (2)	−0.0007 (2)
W4	0.0179 (2)	0.0180 (3)	0.0206 (3)	0.0005 (2)	−0.0041 (2)	−0.0030 (2)
W5	0.0133 (2)	0.0288 (3)	0.0182 (3)	0.0017 (2)	−0.0066 (2)	−0.0074 (2)
W6	0.0148 (2)	0.0254 (3)	0.0188 (3)	0.0014 (2)	−0.0010 (2)	−0.0072 (2)
W7	0.0135 (2)	0.0253 (3)	0.0223 (3)	0.0003 (2)	−0.0071 (2)	0.0000 (2)
W8	0.0151 (2)	0.0274 (3)	0.0172 (3)	0.0002 (2)	−0.0018 (2)	−0.0087 (2)
W9	0.0120 (2)	0.0280 (3)	0.0180 (3)	−0.0008 (2)	−0.0015 (2)	−0.0032 (2)
W10	0.0153 (2)	0.0233 (3)	0.0171 (3)	0.0016 (2)	−0.0034 (2)	−0.0074 (2)
W11	0.0176 (2)	0.0198 (3)	0.0237 (3)	0.0031 (2)	−0.0043 (2)	−0.0046 (2)
W12	0.0178 (2)	0.0188 (3)	0.0256 (3)	0.0016 (2)	−0.0063 (2)	−0.0040 (2)
W13	0.0108 (2)	0.0297 (3)	0.0216 (3)	0.0021 (2)	−0.0026 (2)	−0.0094 (2)
W14	0.0179 (2)	0.0241 (3)	0.0162 (3)	0.0009 (2)	−0.0023 (2)	0.0007 (2)
W15	0.0185 (2)	0.0189 (3)	0.0214 (3)	−0.0008 (2)	−0.0041 (2)	−0.0042 (2)
W16	0.0200 (2)	0.0256 (3)	0.0167 (3)	−0.0019 (2)	−0.0043 (2)	0.0009 (2)
W17	0.0141 (2)	0.0283 (3)	0.0237 (3)	−0.0042 (2)	−0.0032 (2)	−0.0032 (2)
W18	0.0132 (2)	0.0249 (3)	0.0224 (3)	−0.0013 (2)	−0.0053 (2)	−0.0059 (2)
P1	0.0077 (13)	0.0150 (15)	0.0080 (15)	0.0001 (11)	−0.0015 (11)	−0.0034 (12)
P2	0.0055 (12)	0.0163 (16)	0.0083 (15)	−0.0005 (11)	−0.0023 (10)	−0.0008 (12)
O1	0.013 (4)	0.020 (5)	0.027 (5)	−0.002 (3)	−0.004 (3)	0.001 (4)
O2	0.012 (4)	0.033 (5)	0.018 (5)	−0.004 (3)	−0.006 (3)	−0.006 (4)
O3	0.014 (4)	0.034 (5)	0.021 (5)	0.001 (3)	−0.005 (3)	−0.009 (4)
O4	0.021 (4)	0.024 (5)	0.025 (5)	−0.006 (3)	−0.003 (3)	−0.011 (4)
O5	0.009 (4)	0.039 (5)	0.019 (5)	0.001 (3)	0.000 (3)	−0.006 (4)
O6	0.015 (4)	0.025 (5)	0.017 (5)	−0.009 (3)	−0.003 (3)	0.003 (4)
O7	0.024 (4)	0.030 (5)	0.020 (5)	−0.007 (4)	−0.001 (4)	−0.005 (4)
O8	0.027 (5)	0.048 (6)	0.022 (5)	−0.001 (4)	0.002 (4)	−0.016 (4)
O9	0.035 (5)	0.037 (6)	0.026 (5)	−0.010 (4)	−0.012 (4)	0.001 (4)
O10	0.026 (4)	0.039 (5)	0.021 (5)	0.001 (4)	−0.005 (4)	−0.017 (4)
O11	0.019 (4)	0.021 (5)	0.019 (5)	0.002 (3)	0.001 (3)	−0.011 (4)
O12	0.031 (5)	0.042 (6)	0.031 (6)	−0.008 (4)	−0.004 (4)	0.004 (4)
O13	0.019 (4)	0.022 (5)	0.021 (5)	−0.001 (3)	−0.007 (3)	0.000 (4)
O14	0.015 (4)	0.027 (5)	0.018 (5)	0.005 (3)	0.000 (3)	−0.002 (4)
O15	0.032 (4)	0.021 (5)	0.022 (5)	−0.008 (4)	−0.013 (4)	−0.001 (4)
O16	0.015 (4)	0.040 (5)	0.026 (5)	−0.003 (4)	0.000 (4)	−0.007 (4)
O17	0.015 (4)	0.026 (5)	0.019 (5)	0.000 (3)	−0.005 (3)	0.009 (4)
O18	0.016 (4)	0.027 (5)	0.024 (5)	−0.003 (3)	−0.005 (3)	−0.003 (4)
O19	0.029 (5)	0.030 (5)	0.025 (5)	−0.001 (4)	−0.001 (4)	0.004 (4)
O20	0.013 (4)	0.033 (5)	0.019 (5)	−0.004 (3)	0.000 (3)	−0.001 (4)
O21	0.015 (4)	0.024 (5)	0.017 (5)	0.003 (3)	−0.003 (3)	−0.007 (3)
O22	0.019 (4)	0.018 (4)	0.019 (5)	0.006 (3)	−0.001 (3)	−0.002 (3)
O23	0.013 (4)	0.029 (5)	0.017 (5)	−0.002 (3)	−0.001 (3)	−0.006 (4)
O24	0.016 (4)	0.020 (5)	0.027 (5)	0.000 (3)	−0.004 (3)	−0.004 (4)
O25	0.016 (4)	0.032 (5)	0.025 (5)	0.002 (3)	−0.008 (3)	−0.004 (4)
O26	0.009 (4)	0.024 (5)	0.025 (5)	0.001 (3)	−0.007 (3)	0.000 (4)
O27	0.013 (4)	0.028 (5)	0.012 (4)	−0.003 (3)	−0.008 (3)	−0.007 (3)
O28	0.009 (4)	0.023 (5)	0.023 (5)	0.001 (3)	0.000 (3)	−0.002 (4)
O29	0.018 (4)	0.024 (5)	0.014 (4)	0.001 (3)	0.002 (3)	−0.009 (3)
O30	0.012 (4)	0.021 (5)	0.034 (5)	0.007 (3)	−0.003 (3)	−0.013 (4)
O31	0.016 (4)	0.024 (5)	0.021 (5)	−0.003 (3)	0.000 (3)	−0.011 (4)
O32	0.032 (5)	0.027 (5)	0.032 (6)	0.007 (4)	−0.003 (4)	−0.006 (4)
O33	0.016 (4)	0.021 (5)	0.031 (5)	0.005 (3)	−0.008 (3)	−0.008 (4)
O34	0.007 (4)	0.023 (5)	0.024 (5)	0.001 (3)	−0.004 (3)	0.005 (4)
O35	0.015 (4)	0.028 (5)	0.020 (5)	−0.006 (3)	−0.004 (3)	0.003 (4)
O36	0.013 (4)	0.024 (5)	0.015 (4)	0.000 (3)	−0.003 (3)	0.003 (4)
O37	0.021 (4)	0.021 (5)	0.014 (4)	−0.002 (3)	−0.006 (3)	0.002 (3)
O38	0.022 (4)	0.014 (4)	0.044 (6)	−0.004 (3)	−0.004 (4)	−0.004 (4)
O39	0.010 (4)	0.023 (4)	0.013 (4)	0.004 (3)	−0.007 (3)	−0.007 (3)
O40	0.011 (3)	0.025 (3)	0.011 (3)	0.003 (3)	−0.008 (3)	−0.001 (3)
O41	0.016 (4)	0.029 (5)	0.023 (5)	0.001 (3)	−0.007 (3)	−0.009 (4)
O42	0.023 (4)	0.035 (5)	0.032 (6)	−0.014 (4)	−0.007 (4)	0.001 (4)
O43	0.014 (4)	0.044 (6)	0.020 (5)	0.004 (4)	0.002 (3)	−0.003 (4)
O44	0.015 (4)	0.023 (5)	0.025 (5)	0.004 (3)	−0.008 (3)	−0.003 (4)
O45	0.020 (4)	0.020 (5)	0.026 (5)	0.000 (3)	−0.008 (3)	−0.011 (4)
O46	0.010 (4)	0.035 (5)	0.020 (5)	0.001 (3)	−0.006 (3)	−0.006 (4)
O47	0.028 (4)	0.020 (5)	0.021 (5)	0.004 (3)	−0.003 (4)	0.001 (4)
O48	0.022 (4)	0.028 (5)	0.024 (5)	−0.001 (4)	−0.010 (4)	0.004 (4)
O49	0.021 (4)	0.029 (5)	0.027 (5)	−0.005 (4)	−0.002 (4)	−0.006 (4)
O50	0.014 (4)	0.033 (5)	0.011 (4)	−0.003 (3)	−0.002 (3)	−0.004 (4)
O51	0.024 (4)	0.041 (6)	0.024 (5)	0.000 (4)	0.003 (4)	−0.007 (4)
O52	0.034 (5)	0.029 (5)	0.033 (6)	−0.003 (4)	−0.009 (4)	0.003 (4)
O53	0.023 (4)	0.033 (5)	0.029 (5)	0.003 (4)	−0.004 (4)	−0.009 (4)
O54	0.024 (4)	0.036 (5)	0.029 (5)	0.001 (4)	−0.002 (4)	−0.010 (4)
O55	0.012 (4)	0.028 (5)	0.036 (6)	0.000 (3)	−0.010 (4)	−0.002 (4)
O56	0.021 (4)	0.045 (6)	0.031 (6)	0.011 (4)	−0.009 (4)	−0.014 (4)
O57	0.026 (4)	0.028 (5)	0.029 (5)	−0.003 (4)	−0.015 (4)	−0.002 (4)
O58	0.013 (4)	0.025 (5)	0.031 (5)	0.002 (3)	−0.004 (3)	−0.003 (4)
O59	0.018 (4)	0.021 (5)	0.026 (5)	−0.002 (3)	−0.002 (3)	−0.013 (4)
O60	0.030 (5)	0.018 (5)	0.048 (6)	−0.001 (4)	−0.007 (4)	−0.003 (4)
O65	0.016 (4)	0.017 (4)	0.021 (5)	−0.002 (3)	−0.005 (3)	0.004 (4)
O66	0.018 (4)	0.034 (5)	0.047 (6)	0.003 (4)	−0.013 (4)	0.003 (5)
Co2	0.0374 (10)	0.0298 (10)	0.0202 (10)	0.0058 (8)	−0.0065 (8)	−0.0052 (8)
O61	0.036 (5)	0.051 (6)	0.014 (5)	0.018 (4)	−0.010 (4)	0.001 (4)
O62	0.038 (5)	0.029 (5)	0.021 (5)	0.001 (4)	−0.015 (4)	0.001 (4)
O63	0.027 (5)	0.052 (7)	0.039 (6)	0.001 (4)	0.005 (4)	0.009 (5)
O64	0.034 (5)	0.055 (7)	0.049 (7)	0.008 (5)	−0.016 (5)	0.011 (5)
N1	0.047 (7)	0.045 (8)	0.031 (8)	0.018 (6)	0.007 (6)	−0.008 (6)
N2	0.033 (6)	0.026 (6)	0.041 (8)	0.004 (5)	−0.013 (5)	−0.011 (5)
N3	0.039 (6)	0.046 (8)	0.029 (7)	0.007 (5)	−0.012 (5)	−0.010 (6)
N4	0.057 (7)	0.031 (7)	0.040 (8)	0.011 (6)	−0.023 (6)	−0.017 (6)
C1	0.028 (7)	0.039 (8)	0.028 (8)	0.003 (6)	−0.018 (6)	−0.002 (6)
C2	0.039 (6)	0.032 (6)	0.032 (7)	0.003 (5)	−0.021 (6)	−0.015 (5)
C3	0.046 (9)	0.052 (10)	0.039 (10)	−0.003 (7)	0.018 (8)	−0.003 (8)
C4	0.067 (11)	0.047 (11)	0.057 (13)	0.010 (8)	0.029 (9)	0.018 (9)
C5	0.072 (11)	0.031 (8)	0.025 (8)	0.017 (7)	−0.001 (7)	−0.018 (6)
C6	0.073 (11)	0.049 (10)	0.037 (10)	0.005 (8)	−0.019 (8)	−0.022 (8)
Co1	0.0241 (8)	0.0266 (10)	0.0196 (10)	0.0023 (7)	−0.0039 (7)	−0.0031 (7)
O100	0.038 (5)	0.035 (5)	0.015 (5)	0.002 (4)	−0.015 (4)	−0.001 (4)
O101	0.027 (4)	0.036 (5)	0.020 (5)	0.006 (4)	−0.003 (4)	0.001 (4)
O102	0.041 (5)	0.046 (6)	0.038 (6)	0.020 (5)	0.007 (5)	0.004 (5)
O103	0.041 (5)	0.047 (6)	0.029 (6)	−0.003 (5)	0.002 (4)	0.000 (5)
N7	0.030 (6)	0.039 (7)	0.025 (7)	−0.002 (5)	−0.013 (5)	−0.001 (5)
N8	0.031 (6)	0.036 (7)	0.027 (7)	0.002 (5)	−0.005 (5)	−0.004 (5)
N9	0.029 (6)	0.037 (7)	0.028 (7)	−0.001 (5)	−0.002 (5)	−0.002 (5)
N10	0.036 (6)	0.036 (7)	0.025 (7)	0.005 (5)	−0.010 (5)	−0.008 (5)
C9	0.057 (9)	0.034 (9)	0.035 (9)	−0.001 (7)	−0.002 (7)	0.002 (7)
C10	0.042 (8)	0.012 (7)	0.028 (8)	0.009 (5)	0.010 (6)	−0.006 (6)
C11	0.034 (8)	0.055 (10)	0.033 (9)	−0.013 (7)	0.000 (6)	−0.015 (7)
C12	0.023 (6)	0.027 (8)	0.036 (9)	0.006 (5)	0.004 (6)	0.011 (6)
C13	0.032 (7)	0.021 (7)	0.030 (8)	0.004 (6)	0.000 (6)	−0.012 (6)
C14	0.038 (8)	0.034 (8)	0.013 (7)	−0.009 (6)	0.000 (6)	−0.006 (6)
N5	0.051 (7)	0.032 (7)	0.032 (8)	−0.006 (5)	−0.002 (6)	−0.001 (5)
C7	0.039 (8)	0.029 (8)	0.050 (10)	−0.006 (6)	−0.015 (7)	0.002 (7)
O1W	0.109 (10)	0.134 (13)	0.083 (11)	−0.069 (9)	−0.058 (8)	0.042 (10)
O2W	0.093 (10)	0.176 (17)	0.105 (14)	−0.003 (10)	−0.040 (9)	−0.057 (11)
O3W	0.189 (16)	0.124 (14)	0.080 (12)	0.038 (12)	0.033 (12)	−0.008 (10)
O4W	0.088 (10)	0.148 (15)	0.131 (15)	−0.044 (10)	0.053 (10)	−0.074 (11)
O5W	0.085 (10)	0.24 (2)	0.130 (16)	−0.062 (12)	−0.071 (10)	0.080 (15)
O6W	0.167 (19)	0.21 (3)	0.55 (6)	0.080 (18)	0.22 (3)	0.16 (3)
O7W	0.195 (19)	0.19 (2)	0.139 (19)	0.055 (16)	−0.043 (15)	−0.055 (15)
Na1	0.020 (3)	0.019 (3)	0.017 (4)	−0.003 (3)	−0.011 (3)	0.000 (3)

### Geometric parameters (Å, º)

**Table:**

W1—O2	1.921 (6)	W18—O2	1.905 (7)
W1—O3	1.900 (7)	W18—O13	1.887 (8)
W1—O10	1.710 (9)	W18—O15	1.921 (9)
W1—O11	1.908 (7)	W18—O34	2.394 (7)
W1—O37	1.900 (7)	W18—O35	1.931 (7)
W1—O50	2.374 (8)	W18—O53	1.724 (8)
W2—O20	1.883 (7)	Co2—N1	1.934 (11)
W2—O23	1.895 (8)	Co2—O61	1.919 (8)
W2—O24	1.920 (7)	Co2—O62	1.889 (9)
W2—O28	2.376 (7)	Co2—N4	1.960 (11)
W2—O44	1.945 (7)	Co2—N2	1.955 (10)
W2—O52	1.705 (9)	Co2—N3	1.961 (11)
W3—O4	1.907 (7)	Co1—N10	1.950 (11)
W3—O6	1.931 (6)	Co1—N9	1.948 (10)
W3—O7	1.906 (8)	Co1—N8	1.938 (10)
W3—O9	1.688 (9)	Co1—O100	1.922 (9)
W3—O27	2.408 (7)	Co1—O101	1.927 (8)
W3—O59	1.916 (7)	Co1—N7	1.936 (10)
W4—O22	1.908 (7)	P1—O28	1.542 (8)
W4—O35	1.902 (6)	P1—O39	1.590 (6)
W4—O36	2.344 (7)	P1—O27	1.525 (8)
W4—O37	1.895 (7)	P1—O26	1.533 (8)
W4—O38	1.703 (8)	P2—O34	1.569 (6)
W4—O46	1.920 (6)	P2—O50	1.528 (8)
W5—O39	2.378 (7)	P2—O65	1.517 (7)
W5—O40	1.922 (7)	P2—O36	1.549 (7)
W5—O43	1.720 (7)	O61—C1	1.290 (16)
W5—O44	1.892 (8)	O62—C2	1.282 (15)
W5—O45	1.909 (7)	O63—C2	1.239 (16)
W5—O57	1.922 (8)	O64—C1	1.227 (15)
W6—O18	1.915 (8)	O100—C13	1.288 (14)
W6—O21	1.895 (6)	O101—C14	1.262 (15)
W6—O33	1.910 (9)	O102—C14	1.241 (16)
W6—O47	1.907 (8)	O103—C13	1.217 (16)
W6—O51	1.714 (9)	O1W—H1WA	0.8500
W6—O65	2.385 (7)	O1W—H1WB	0.8500
W7—O1	1.889 (7)	O2W—H2WA	0.8500
W7—O13	1.972 (8)	O2W—H2WB	0.8500
W7—O14	1.930 (7)	N1—C4	1.48 (2)
W7—O30	1.882 (8)	N2—C3	1.480 (18)
W7—O34	2.385 (7)	N3—C5	1.472 (18)
W7—O66	1.697 (8)	N4—C6	1.494 (18)
W8—O3	1.901 (7)	N1—H1A	0.9000
W8—O5	1.923 (6)	N1—H1B	0.9000
W8—O7	1.916 (8)	N2—H2B	0.9000
W8—O8	1.707 (9)	N2—H2A	0.9000
W8—O27	2.348 (7)	N3—H3D	0.9000
W8—O29	1.890 (7)	N3—H3C	0.9000
W9—O14	1.895 (8)	O3W—H3WB	0.8500
W9—O15	1.916 (9)	O3W—H3WA	0.8500
W9—O16	1.719 (8)	N4—H4D	0.9000
W9—O17	1.936 (7)	N4—H4C	0.9000
W9—O18	1.931 (8)	N7—C9	1.466 (16)
W9—O34	2.398 (7)	N8—C10	1.466 (16)
W10—O21	1.898 (6)	N9—C11	1.477 (17)
W10—O23	1.892 (8)	N10—C12	1.496 (17)
W10—O25	1.920 (9)	O4W—H4WA	0.8500
W10—O26	2.374 (7)	O4W—H4WB	0.8500
W10—O45	1.946 (7)	N7—H7C	0.9000
W10—O54	1.713 (9)	N7—H7D	0.9000
W11—O24	1.918 (7)	N8—H8E	0.9000
W11—O28	2.348 (8)	N8—H8D	0.9000
W11—O29	1.902 (7)	N9—H9C	0.9000
W11—O46	1.880 (6)	N9—H9D	0.9000
W11—O55	1.940 (8)	N10—H10D	0.9000
W11—O60	1.702 (8)	N10—H10C	0.9000
W12—O30	1.946 (8)	N5—C7	1.432 (18)
W12—O31	1.882 (7)	N5—H8C	0.8900
W12—O32	1.697 (8)	N5—H8A	0.8900
W12—O33	1.925 (9)	N5—H8B	0.8900
W12—O48	1.878 (9)	O5W—H5WB	0.8500
W12—O65	2.378 (7)	O5W—H5WA	0.8500
W13—O5	1.926 (7)	O6W—H6WD	0.8500
W13—O39	2.374 (6)	O6W—H6WC	0.8500
W13—O40	1.925 (7)	O7W—H7WA	0.8500
W13—O41	1.947 (8)	O7W—H7WB	0.8500
W13—O55	1.880 (8)	C1—C2	1.569 (19)
W13—O56	1.713 (8)	C3—C4	1.49 (2)
W14—O17	1.905 (6)	C5—C6	1.50 (2)
W14—O19	1.711 (9)	C3—H3A	0.9700
W14—O20	1.905 (7)	C3—H3B	0.9700
W14—O22	1.915 (7)	C4—H4A	0.9700
W14—O36	2.362 (7)	C4—H4B	0.9700
W14—O47	1.894 (8)	C5—H5B	0.9700
W15—O25	1.933 (9)	C5—H5A	0.9700
W15—O26	2.360 (8)	C6—H6A	0.9700
W15—O31	1.929 (7)	C6—H6B	0.9700
W15—O49	1.710 (8)	C9—C10	1.492 (18)
W15—O58	1.901 (7)	C11—C12	1.484 (19)
W15—O59	1.889 (7)	C13—C14	1.542 (18)
W16—O1	1.931 (6)	C9—H9B	0.9700
W16—O4	1.888 (7)	C9—H9A	0.9700
W16—O11	1.926 (7)	C10—H10A	0.9700
W16—O12	1.675 (9)	C10—H10B	0.9700
W16—O48	1.903 (9)	C11—H11B	0.9700
W16—O50	2.378 (8)	C11—H11A	0.9700
W17—O6	1.884 (7)	C12—H12B	0.9700
W17—O39	2.383 (7)	C12—H12A	0.9700
W17—O41	1.888 (8)	C7—C7^i^	1.576 (19)
W17—O42	1.697 (8)	C7—H7B	0.9700
W17—O57	1.927 (9)	C7—H7A	0.9700
W17—O58	1.935 (8)		
			
O2—W1—O3	164.0 (3)	O2—W18—O13	91.1 (3)
O2—W1—O10	96.5 (3)	O2—W18—O15	156.0 (3)
O2—W1—O11	90.0 (3)	O2—W18—O34	83.9 (3)
O2—W1—O37	85.2 (3)	O2—W18—O35	84.3 (3)
O2—W1—O50	81.8 (3)	O2—W18—O53	101.3 (4)
O3—W1—O10	99.3 (3)	O13—W18—O15	87.8 (3)
O3—W1—O11	89.7 (3)	O13—W18—O34	73.6 (3)
O3—W1—O37	88.9 (3)	O13—W18—O35	156.6 (3)
O3—W1—O50	82.8 (3)	O13—W18—O53	101.9 (4)
O10—W1—O11	100.5 (4)	O15—W18—O34	72.8 (3)
O10—W1—O37	102.2 (4)	O15—W18—O35	87.2 (3)
O10—W1—O50	173.3 (3)	O15—W18—O53	102.4 (4)
O11—W1—O37	157.1 (3)	O34—W18—O35	83.1 (3)
O11—W1—O50	73.0 (3)	O34—W18—O53	173.3 (3)
O37—W1—O50	84.2 (3)	O35—W18—O53	101.5 (4)
O20—W2—O23	89.7 (3)	O61—Co2—O62	85.8 (4)
O20—W2—O24	90.7 (3)	O61—Co2—N1	89.2 (4)
O20—W2—O28	82.3 (3)	O61—Co2—N2	89.8 (4)
O20—W2—O44	163.3 (3)	O61—Co2—N3	174.8 (4)
O20—W2—O52	99.0 (3)	O61—Co2—N4	91.7 (4)
O23—W2—O24	157.1 (3)	O62—Co2—N1	173.8 (4)
O23—W2—O28	84.3 (3)	O62—Co2—N2	90.6 (4)
O23—W2—O44	84.6 (3)	O62—Co2—N3	90.3 (4)
O23—W2—O52	102.9 (4)	O62—Co2—N4	90.0 (4)
O24—W2—O28	73.1 (3)	N1—Co2—N2	85.8 (5)
O24—W2—O44	88.6 (3)	N1—Co2—N3	94.9 (5)
O24—W2—O52	99.6 (4)	N1—Co2—N4	93.7 (5)
O28—W2—O44	81.6 (3)	N2—Co2—N3	93.7 (4)
O28—W2—O52	172.6 (3)	N2—Co2—N4	178.4 (5)
O44—W2—O52	97.5 (3)	N3—Co2—N4	84.8 (5)
O4—W3—O6	161.3 (3)	N7—Co1—N8	85.6 (4)
O4—W3—O7	90.6 (3)	N7—Co1—N9	175.8 (4)
O4—W3—O9	99.1 (3)	N7—Co1—N10	93.4 (4)
O4—W3—O27	81.8 (3)	N8—Co1—N9	90.4 (4)
O4—W3—O59	87.3 (3)	N8—Co1—N10	95.4 (4)
O6—W3—O7	89.7 (3)	N9—Co1—N10	86.0 (4)
O6—W3—O9	99.3 (3)	O100—Co1—N10	173.4 (4)
O6—W3—O27	80.5 (3)	O101—Co1—N7	92.9 (4)
O6—W3—O59	85.3 (3)	O101—Co1—N10	88.6 (4)
O7—W3—O9	100.9 (4)	O100—Co1—O101	85.5 (4)
O7—W3—O27	72.6 (3)	O100—Co1—N7	89.9 (4)
O7—W3—O59	157.4 (3)	O100—Co1—N8	90.6 (4)
O9—W3—O27	173.5 (3)	O100—Co1—N9	91.1 (4)
O9—W3—O59	101.7 (4)	O101—Co1—N9	91.2 (4)
O27—W3—O59	84.8 (3)	O101—Co1—N8	175.9 (4)
O22—W4—O35	90.5 (3)	O26—P1—O39	107.2 (4)
O22—W4—O36	73.0 (3)	O28—P1—O39	107.0 (4)
O22—W4—O37	158.2 (3)	O26—P1—O28	112.1 (4)
O22—W4—O38	98.7 (4)	O26—P1—O27	111.8 (4)
O22—W4—O46	89.5 (3)	O27—P1—O28	111.0 (4)
O35—W4—O36	82.4 (3)	O27—P1—O39	107.4 (4)
O35—W4—O37	86.0 (3)	O34—P2—O65	107.3 (4)
O35—W4—O38	98.7 (4)	O36—P2—O65	112.0 (4)
O35—W4—O46	163.6 (4)	O34—P2—O50	106.9 (4)
O36—W4—O37	85.2 (3)	O34—P2—O36	106.5 (4)
O36—W4—O38	171.7 (3)	O36—P2—O50	112.0 (4)
O36—W4—O46	81.9 (3)	O50—P2—O65	111.8 (4)
O37—W4—O38	103.1 (4)	W7—O1—W16	151.8 (4)
O37—W4—O46	88.0 (3)	W1—O2—W18	152.5 (4)
O38—W4—O46	97.5 (4)	W1—O3—W8	162.5 (4)
O39—W5—O40	73.4 (3)	W3—O4—W16	164.1 (5)
O39—W5—O43	171.0 (3)	W8—O5—W13	149.6 (4)
O39—W5—O44	84.8 (3)	W3—O6—W17	154.4 (4)
O39—W5—O45	84.1 (3)	W3—O7—W8	123.4 (4)
O39—W5—O57	72.1 (3)	W1—O11—W16	123.6 (4)
O40—W5—O43	101.9 (3)	W7—O13—W18	123.4 (4)
O40—W5—O44	89.5 (3)	W7—O14—W9	123.9 (4)
O40—W5—O45	157.4 (3)	W9—O15—W18	124.2 (4)
O40—W5—O57	87.4 (3)	W9—O17—W14	151.2 (4)
O43—W5—O44	103.0 (3)	W6—O18—W9	151.0 (5)
O43—W5—O45	100.7 (3)	W2—O20—W14	162.8 (5)
O43—W5—O57	100.2 (4)	W6—O21—W10	162.6 (4)
O44—W5—O45	86.2 (3)	W4—O22—W14	123.2 (4)
O44—W5—O57	156.7 (3)	W2—O23—W10	153.6 (4)
O45—W5—O57	87.8 (3)	W2—O24—W11	122.2 (4)
O18—W6—O21	164.5 (3)	W10—O25—W15	121.9 (4)
O18—W6—O33	90.5 (3)	W15—O26—P1	127.9 (4)
O18—W6—O47	86.1 (3)	W10—O26—P1	127.7 (4)
O18—W6—O51	97.4 (3)	W10—O26—W15	90.7 (3)
O18—W6—O65	83.2 (3)	W3—O27—P1	127.2 (4)
O21—W6—O33	89.4 (3)	W3—O27—W8	90.1 (2)
O21—W6—O47	88.1 (3)	W8—O27—P1	128.7 (4)
O21—W6—O51	97.9 (3)	W11—O28—P1	127.9 (4)
O21—W6—O65	81.9 (3)	W2—O28—W11	90.7 (3)
O33—W6—O47	157.6 (3)	W2—O28—P1	128.0 (4)
O33—W6—O51	100.6 (4)	W8—O29—W11	152.5 (4)
O33—W6—O65	73.2 (3)	W7—O30—W12	150.8 (5)
O47—W6—O51	101.8 (4)	W12—O31—W15	161.3 (4)
O47—W6—O65	84.4 (3)	W6—O33—W12	123.3 (4)
O51—W6—O65	173.8 (3)	W7—O34—W9	89.8 (2)
O1—W7—O13	88.8 (3)	W7—O34—W18	90.6 (2)
O1—W7—O14	156.0 (3)	W7—O34—P2	124.4 (4)
O1—W7—O30	86.3 (3)	W18—O34—P2	125.6 (4)
O1—W7—O34	82.9 (3)	W9—O34—P2	125.5 (4)
O1—W7—O66	102.9 (3)	W9—O34—W18	90.1 (2)
O13—W7—O14	85.3 (3)	W4—O35—W18	151.5 (4)
O13—W7—O30	157.2 (3)	W4—O36—W14	91.2 (2)
O13—W7—O34	72.4 (3)	W14—O36—P2	127.8 (4)
O13—W7—O66	100.4 (4)	W4—O36—P2	127.7 (4)
O14—W7—O30	90.1 (4)	W1—O37—W4	151.4 (4)
O14—W7—O34	73.1 (3)	W5—O39—W13	90.5 (2)
O14—W7—O66	101.2 (4)	W17—O39—P1	124.8 (4)
O30—W7—O34	84.9 (3)	W13—O39—W17	90.3 (2)
O30—W7—O66	102.4 (4)	W5—O39—W17	91.4 (2)
O34—W7—O66	170.9 (3)	W5—O39—P1	124.5 (4)
O3—W8—O5	165.1 (3)	W13—O39—P1	125.0 (4)
O3—W8—O7	90.3 (3)	W5—O40—W13	122.6 (3)
O3—W8—O8	97.6 (3)	W13—O41—W17	123.2 (4)
O3—W8—O27	83.0 (3)	W2—O44—W5	151.3 (5)
O3—W8—O29	88.1 (3)	W5—O45—W10	150.1 (4)
O5—W8—O7	89.3 (3)	W4—O46—W11	162.9 (4)
O5—W8—O8	97.2 (3)	W6—O47—W14	150.4 (4)
O5—W8—O27	82.6 (3)	W12—O48—W16	152.9 (5)
O5—W8—O29	86.5 (3)	W16—O50—P2	127.9 (4)
O7—W8—O8	99.7 (4)	W1—O50—P2	128.2 (5)
O7—W8—O27	73.9 (3)	W1—O50—W16	90.6 (2)
O7—W8—O29	157.1 (3)	W11—O55—W13	152.5 (5)
O8—W8—O27	173.6 (3)	W5—O57—W17	124.5 (4)
O8—W8—O29	103.1 (4)	W15—O58—W17	150.0 (5)
O27—W8—O29	83.3 (3)	W3—O59—W15	149.6 (4)
O14—W9—O15	88.9 (3)	W6—O65—W12	90.2 (2)
O14—W9—O16	103.1 (4)	W6—O65—P2	128.0 (4)
O14—W9—O17	156.7 (3)	W12—O65—P2	128.8 (4)
O14—W9—O18	90.0 (3)	Co2—O61—C1	113.0 (8)
O14—W9—O34	73.3 (3)	Co2—O62—C2	114.3 (8)
O15—W9—O16	102.2 (4)	Co1—O100—C13	112.7 (8)
O15—W9—O17	87.7 (3)	Co1—O101—C14	111.9 (8)
O15—W9—O18	156.9 (3)	H1WA—O1W—H1WB	113.00
O15—W9—O34	72.8 (3)	H2WA—O2W—H2WB	108.00
O16—W9—O17	100.2 (3)	Co2—N1—C4	109.9 (9)
O16—W9—O18	100.5 (4)	Co2—N2—C3	111.0 (9)
O16—W9—O34	173.6 (3)	Co2—N3—C5	111.4 (8)
O17—W9—O18	84.3 (3)	Co2—N4—C6	108.4 (9)
O17—W9—O34	83.7 (3)	C4—N1—H1A	110.00
O18—W9—O34	84.9 (3)	H1A—N1—H1B	108.00
O21—W10—O23	89.3 (3)	Co2—N1—H1A	110.00
O21—W10—O25	90.8 (3)	Co2—N1—H1B	110.00
O21—W10—O26	82.7 (3)	C4—N1—H1B	110.00
O21—W10—O45	163.7 (3)	C3—N2—H2A	109.00
O21—W10—O54	99.8 (3)	Co2—N2—H2B	109.00
O23—W10—O25	158.1 (3)	Co2—N2—H2A	109.00
O23—W10—O26	84.8 (3)	H2A—N2—H2B	108.00
O23—W10—O45	85.2 (3)	C3—N2—H2B	110.00
O23—W10—O54	102.1 (4)	H3C—N3—H3D	108.00
O25—W10—O26	73.6 (3)	C5—N3—H3C	109.00
O25—W10—O45	88.7 (3)	C5—N3—H3D	109.00
O25—W10—O54	99.4 (4)	Co2—N3—H3C	109.00
O26—W10—O45	81.5 (3)	Co2—N3—H3D	109.00
O26—W10—O54	172.7 (3)	H3WA—O3W—H3WB	108.00
O45—W10—O54	96.4 (3)	Co2—N4—H4D	110.00
O24—W11—O28	73.8 (3)	H4C—N4—H4D	108.00
O24—W11—O29	157.4 (3)	Co2—N4—H4C	110.00
O24—W11—O46	90.3 (3)	C6—N4—H4D	110.00
O24—W11—O55	89.4 (3)	C6—N4—H4C	110.00
O24—W11—O60	99.7 (4)	Co1—N7—C9	110.7 (8)
O28—W11—O29	83.8 (3)	Co1—N8—C10	109.1 (7)
O28—W11—O46	82.2 (3)	Co1—N9—C11	110.6 (8)
O28—W11—O55	81.5 (3)	Co1—N10—C12	106.3 (8)
O28—W11—O60	173.4 (3)	H4WA—O4W—H4WB	108.00
O29—W11—O46	88.7 (3)	H7C—N7—H7D	108.00
O29—W11—O55	85.1 (3)	Co1—N7—H7D	110.00
O29—W11—O60	102.7 (4)	C9—N7—H7C	110.00
O46—W11—O55	163.1 (4)	C9—N7—H7D	110.00
O46—W11—O60	99.2 (4)	Co1—N7—H7C	110.00
O55—W11—O60	97.4 (3)	H8D—N8—H8E	108.00
O30—W12—O31	164.3 (3)	Co1—N8—H8E	110.00
O30—W12—O32	96.6 (4)	Co1—N8—H8D	110.00
O30—W12—O33	88.2 (3)	C10—N8—H8E	110.00
O30—W12—O48	85.1 (3)	C10—N8—H8D	110.00
O30—W12—O65	81.1 (3)	C11—N9—H9D	110.00
O31—W12—O32	99.0 (4)	H9C—N9—H9D	108.00
O31—W12—O33	90.5 (3)	C11—N9—H9C	109.00
O31—W12—O48	90.2 (3)	Co1—N9—H9D	110.00
O31—W12—O65	83.6 (3)	Co1—N9—H9C	110.00
O32—W12—O33	99.1 (4)	Co1—N10—H10D	110.00
O32—W12—O48	103.1 (4)	C12—N10—H10C	110.00
O32—W12—O65	171.9 (3)	Co1—N10—H10C	110.00
O33—W12—O48	157.4 (4)	C12—N10—H10D	110.00
O33—W12—O65	73.1 (3)	H10C—N10—H10D	109.00
O48—W12—O65	84.6 (3)	H8A—N5—H8C	109.00
O5—W13—O39	84.0 (3)	C7—N5—H8C	109.00
O5—W13—O40	157.3 (3)	H8A—N5—H8B	109.00
O5—W13—O41	87.1 (3)	C7—N5—H8B	109.00
O5—W13—O55	86.1 (3)	H8B—N5—H8C	109.00
O5—W13—O56	101.6 (4)	C7—N5—H8A	110.00
O39—W13—O40	73.5 (2)	H5WA—O5W—H5WB	109.00
O39—W13—O41	72.8 (3)	H6WC—O6W—H6WD	108.00
O39—W13—O55	83.4 (3)	H7WA—O7W—H7WB	108.00
O39—W13—O56	172.8 (3)	O64—C1—C2	121.5 (12)
O40—W13—O41	88.6 (3)	O61—C1—O64	125.0 (13)
O40—W13—O55	88.8 (3)	O61—C1—C2	113.4 (10)
O40—W13—O56	101.1 (3)	O63—C2—C1	120.1 (11)
O41—W13—O55	155.9 (3)	O62—C2—C1	113.4 (11)
O41—W13—O56	102.6 (4)	O62—C2—O63	126.6 (12)
O55—W13—O56	101.4 (4)	N2—C3—C4	108.8 (12)
O17—W14—O19	98.7 (3)	N1—C4—C3	108.6 (13)
O17—W14—O20	163.3 (3)	N3—C5—C6	105.8 (11)
O17—W14—O22	89.9 (3)	N4—C6—C5	107.3 (11)
O17—W14—O36	82.3 (3)	C4—C3—H3B	110.00
O17—W14—O47	87.0 (3)	N2—C3—H3B	110.00
O19—W14—O20	97.9 (3)	H3A—C3—H3B	108.00
O19—W14—O22	100.5 (4)	C4—C3—H3A	110.00
O19—W14—O36	173.0 (3)	N2—C3—H3A	110.00
O19—W14—O47	102.2 (4)	H4A—C4—H4B	108.00
O20—W14—O22	89.1 (3)	C3—C4—H4A	110.00
O20—W14—O36	81.5 (3)	C3—C4—H4B	110.00
O20—W14—O47	87.5 (3)	N1—C4—H4B	110.00
O22—W14—O36	72.5 (3)	N1—C4—H4A	110.00
O22—W14—O47	157.3 (3)	N3—C5—H5B	111.00
O36—W14—O47	84.8 (3)	H5A—C5—H5B	109.00
O25—W15—O26	73.7 (3)	C6—C5—H5B	111.00
O25—W15—O31	88.5 (3)	C6—C5—H5A	111.00
O25—W15—O49	98.9 (4)	N3—C5—H5A	111.00
O25—W15—O58	89.3 (3)	H6A—C6—H6B	108.00
O25—W15—O59	159.1 (3)	C5—C6—H6B	110.00
O26—W15—O31	82.1 (3)	N4—C6—H6B	110.00
O26—W15—O49	172.5 (3)	C5—C6—H6A	110.00
O26—W15—O58	82.1 (3)	N4—C6—H6A	110.00
O26—W15—O59	85.4 (3)	N7—C9—C10	106.2 (10)
O31—W15—O49	97.0 (3)	N8—C10—C9	108.6 (10)
O31—W15—O58	164.0 (4)	N9—C11—C12	106.6 (10)
O31—W15—O59	88.1 (3)	N10—C12—C11	107.8 (11)
O49—W15—O58	99.0 (4)	O100—C13—C14	113.4 (11)
O49—W15—O59	102.0 (4)	O103—C13—C14	122.7 (11)
O58—W15—O59	88.3 (3)	O100—C13—O103	123.9 (12)
O1—W16—O4	162.4 (3)	O102—C14—C13	118.0 (11)
O1—W16—O11	89.3 (3)	O101—C14—C13	116.3 (11)
O1—W16—O12	97.5 (3)	O101—C14—O102	125.6 (12)
O1—W16—O48	85.1 (3)	H9A—C9—H9B	109.00
O1—W16—O50	80.5 (2)	N7—C9—H9A	110.00
O4—W16—O11	90.2 (3)	C10—C9—H9B	111.00
O4—W16—O12	100.0 (3)	N7—C9—H9B	111.00
O4—W16—O48	88.6 (3)	C10—C9—H9A	111.00
O4—W16—O50	82.7 (3)	C9—C10—H10B	110.00
O11—W16—O12	98.3 (4)	N8—C10—H10A	110.00
O11—W16—O48	157.2 (3)	N8—C10—H10B	110.00
O11—W16—O50	72.6 (3)	H10A—C10—H10B	108.00
O12—W16—O48	104.4 (4)	C9—C10—H10A	110.00
O12—W16—O50	170.6 (3)	C12—C11—H11B	110.00
O48—W16—O50	84.7 (3)	H11A—C11—H11B	109.00
O6—W17—O39	83.5 (3)	C12—C11—H11A	110.00
O6—W17—O41	89.8 (3)	N9—C11—H11B	110.00
O6—W17—O42	103.9 (4)	N9—C11—H11A	110.00
O6—W17—O57	154.9 (3)	H12A—C12—H12B	108.00
O6—W17—O58	85.0 (3)	N10—C12—H12A	110.00
O39—W17—O41	73.6 (3)	N10—C12—H12B	110.00
O39—W17—O42	171.9 (3)	C11—C12—H12A	110.00
O39—W17—O57	71.9 (3)	C11—C12—H12B	110.00
O39—W17—O58	83.3 (3)	N5—C7—C7^i^	112.3 (11)
O41—W17—O42	102.7 (4)	N5—C7—H7A	109.00
O41—W17—O57	88.3 (3)	N5—C7—H7B	109.00
O41—W17—O58	156.7 (3)	H7A—C7—H7B	108.00
O42—W17—O57	101.0 (4)	C7^i^—C7—H7A	109.00
O42—W17—O58	100.6 (4)	C7^i^—C7—H7B	109.00
O57—W17—O58	87.0 (3)		
			
O11—W1—O37—W4	−48.8 (14)	O56—W13—O40—W5	−174.0 (4)
O50—W1—O37—W4	−52.5 (8)	O39—W13—O41—W17	−2.4 (4)
O10—W1—O11—W16	178.4 (4)	O56—W13—O55—W11	127.8 (11)
O37—W1—O11—W16	−7.4 (11)	O41—W13—O40—W5	−71.4 (4)
O50—W1—O11—W16	−3.5 (4)	O39—W13—O40—W5	1.1 (3)
O3—W1—O50—P2	128.3 (5)	O55—W13—O39—P1	42.7 (5)
O11—W1—O50—P2	−139.8 (5)	O56—W13—O5—W8	−127.5 (9)
O37—W1—O50—P2	38.7 (5)	O40—W13—O5—W8	50.9 (15)
O2—W1—O11—W16	−85.0 (5)	O5—W13—O39—W5	−178.2 (3)
O10—W1—O2—W18	−131.8 (10)	O40—W13—O41—W17	70.8 (4)
O11—W1—O2—W18	127.6 (10)	O40—W13—O39—W5	−0.8 (2)
O37—W1—O2—W18	−30.0 (10)	O41—W13—O39—W5	92.9 (3)
O50—W1—O2—W18	54.8 (10)	O39—W13—O5—W8	57.2 (9)
O11—W1—O50—W16	2.4 (3)	O5—W13—O41—W17	−86.9 (4)
O2—W1—O37—W4	29.7 (8)	O41—W13—O55—W11	−47.3 (16)
O2—W1—O50—P2	−47.3 (5)	O41—W13—O5—W8	130.2 (9)
O3—W1—O11—W16	79.1 (5)	O40—W13—O39—P1	133.4 (5)
O10—W1—O37—W4	125.3 (8)	O41—W13—O39—P1	−132.9 (5)
O2—W1—O50—W16	94.9 (3)	O5—W13—O39—P1	−44.1 (5)
O3—W1—O50—W16	−89.5 (3)	O40—W13—O55—W11	−131.2 (11)
O3—W1—O37—W4	−135.4 (9)	O55—W13—O40—W5	84.6 (4)
O37—W1—O50—W16	−179.1 (3)	O39—W13—O55—W11	−57.7 (11)
O23—W2—O44—W5	29.0 (9)	O56—W13—O41—W17	171.8 (5)
O52—W2—O44—W5	131.4 (9)	O55—W13—O41—W17	−13.1 (11)
O20—W2—O28—P1	−128.0 (5)	O55—W13—O5—W8	−26.6 (9)
O44—W2—O24—W11	86.4 (4)	O41—W13—O39—W17	1.6 (3)
O23—W2—O28—P1	−37.5 (5)	O5—W13—O55—W11	26.7 (11)
O24—W2—O44—W5	−129.1 (9)	O55—W13—O39—W5	−91.5 (3)
O28—W2—O44—W5	−56.0 (9)	O5—W13—O40—W5	7.7 (10)
O23—W2—O28—W11	−179.7 (2)	O40—W13—O39—W17	−92.1 (3)
O44—W2—O23—W10	−32.1 (9)	O55—W13—O39—W17	177.1 (3)
O52—W2—O23—W10	−128.6 (9)	O5—W13—O39—W17	90.4 (3)
O28—W2—O23—W10	50.0 (9)	O22—W14—O17—W9	128.6 (9)
O20—W2—O24—W11	−76.9 (5)	O17—W14—O36—P2	−48.4 (5)
O24—W2—O28—P1	138.9 (5)	O36—W14—O17—W9	56.3 (8)
O44—W2—O28—P1	47.8 (5)	O20—W14—O22—W4	79.1 (5)
O24—W2—O28—W11	−3.3 (3)	O17—W14—O22—W4	−84.2 (5)
O20—W2—O28—W11	89.9 (3)	O19—W14—O22—W4	176.9 (4)
O24—W2—O23—W10	41.1 (13)	O47—W14—O36—P2	39.3 (5)
O44—W2—O28—W11	−94.3 (3)	O47—W14—O22—W4	−2.4 (11)
O20—W2—O23—W10	132.2 (9)	O17—W14—O47—W6	28.0 (9)
O52—W2—O24—W11	−176.2 (4)	O47—W14—O36—W4	−178.4 (3)
O28—W2—O24—W11	4.7 (4)	O47—W14—O17—W9	−28.9 (9)
O23—W2—O24—W11	14.0 (10)	O20—W14—O36—P2	127.5 (5)
O59—W3—O7—W8	−6.6 (11)	O22—W14—O36—P2	−140.7 (5)
O9—W3—O7—W8	177.2 (4)	O36—W14—O22—W4	−2.3 (4)
O27—W3—O6—W17	58.3 (11)	O20—W14—O47—W6	−136.2 (9)
O7—W3—O27—W8	2.3 (3)	O22—W14—O47—W6	−54.5 (14)
O27—W3—O7—W8	−3.4 (4)	O36—W14—O47—W6	−54.5 (8)
O59—W3—O6—W17	−27.2 (11)	O22—W14—O36—W4	1.6 (3)
O7—W3—O27—P1	−139.6 (5)	O19—W14—O47—W6	126.3 (8)
O6—W3—O27—P1	−46.9 (5)	O17—W14—O36—W4	94.0 (3)
O9—W3—O59—W15	126.4 (8)	O20—W14—O36—W4	−90.2 (3)
O27—W3—O59—W15	−53.0 (8)	O19—W14—O17—W9	−130.7 (9)
O6—W3—O59—W15	27.9 (8)	O31—W15—O26—P1	−129.6 (5)
O6—W3—O7—W8	−83.5 (5)	O59—W15—O25—W10	1.8 (12)
O7—W3—O6—W17	130.7 (11)	O25—W15—O26—P1	139.6 (5)
O9—W3—O6—W17	−128.3 (11)	O58—W15—O25—W10	85.2 (5)
O4—W3—O27—W8	−90.9 (3)	O59—W15—O58—W17	25.6 (10)
O4—W3—O27—P1	127.2 (5)	O31—W15—O25—W10	−79.0 (5)
O59—W3—O27—P1	39.2 (5)	O58—W15—O26—W10	−93.8 (3)
O6—W3—O27—W8	95.0 (3)	O49—W15—O25—W10	−175.8 (4)
O59—W3—O27—W8	−179.0 (3)	O26—W15—O59—W3	53.5 (8)
O4—W3—O7—W8	77.8 (5)	O49—W15—O58—W17	127.6 (10)
O7—W3—O59—W15	−49.8 (13)	O25—W15—O26—W10	−2.2 (3)
O4—W3—O59—W15	−134.9 (8)	O25—W15—O59—W3	54.8 (14)
O35—W4—O36—P2	48.0 (5)	O58—W15—O26—P1	48.0 (5)
O46—W4—O36—P2	−127.3 (5)	O26—W15—O25—W10	3.2 (4)
O22—W4—O36—P2	140.8 (5)	O26—W15—O58—W17	−59.9 (10)
O36—W4—O22—W14	2.3 (4)	O59—W15—O26—P1	−40.9 (5)
O46—W4—O22—W14	−79.4 (5)	O31—W15—O26—W10	88.6 (3)
O46—W4—O37—W1	135.0 (9)	O49—W15—O59—W3	−127.5 (8)
O38—W4—O35—W18	131.5 (9)	O25—W15—O58—W17	−133.6 (10)
O38—W4—O37—W1	−127.8 (8)	O59—W15—O26—W10	177.3 (3)
O37—W4—O36—P2	−38.6 (5)	O31—W15—O59—W3	135.7 (8)
O37—W4—O22—W14	3.8 (11)	O58—W15—O59—W3	−28.7 (8)
O35—W4—O37—W1	−29.8 (9)	O48—W16—O50—W1	179.5 (3)
O46—W4—O36—W14	90.3 (3)	O4—W16—O50—W1	90.2 (3)
O35—W4—O36—W14	−94.4 (3)	O11—W16—O50—W1	−2.4 (2)
O37—W4—O35—W18	28.8 (9)	O12—W16—O1—W7	127.9 (8)
O37—W4—O36—W14	179.0 (3)	O50—W16—O1—W7	−61.4 (8)
O38—W4—O22—W14	−176.9 (4)	O11—W16—O1—W7	−133.9 (8)
O22—W4—O37—W1	51.5 (14)	O50—W16—O11—W1	3.5 (4)
O22—W4—O36—W14	−1.6 (3)	O48—W16—O1—W7	24.0 (8)
O36—W4—O35—W18	−56.8 (9)	O50—W16—O48—W12	49.9 (10)
O36—W4—O37—W1	52.9 (8)	O11—W16—O48—W12	45.4 (15)
O35—W4—O22—W14	84.3 (5)	O4—W16—O48—W12	132.6 (10)
O22—W4—O35—W18	−129.6 (9)	O1—W16—O48—W12	−31.0 (10)
O45—W5—O39—W13	178.5 (3)	O1—W16—O50—P2	47.8 (5)
O45—W5—O40—W13	−6.9 (10)	O1—W16—O50—W1	−94.7 (3)
O39—W5—O40—W13	−1.1 (3)	O12—W16—O11—W1	−178.8 (5)
O43—W5—O40—W13	170.9 (4)	O12—W16—O48—W12	−127.4 (10)
O57—W5—O40—W13	71.0 (4)	O48—W16—O50—P2	−38.1 (5)
O44—W5—O40—W13	−85.8 (4)	O11—W16—O50—P2	140.1 (5)
O39—W5—O45—W10	−58.3 (8)	O48—W16—O11—W1	8.2 (11)
O57—W5—O44—W2	46.8 (14)	O4—W16—O50—P2	−127.4 (5)
O40—W5—O45—W10	−52.7 (13)	O1—W16—O11—W1	83.7 (5)
O57—W5—O45—W10	−130.5 (8)	O4—W16—O11—W1	−78.7 (5)
O40—W5—O39—W13	0.8 (2)	O57—W17—O39—W5	1.5 (3)
O44—W5—O39—W13	91.8 (3)	O41—W17—O39—W13	−1.6 (3)
O45—W5—O57—W17	86.9 (5)	O41—W17—O6—W3	−131.3 (11)
O57—W5—O39—W13	−91.9 (3)	O6—W17—O39—W5	176.2 (3)
O43—W5—O44—W2	−128.8 (9)	O6—W17—O39—W13	−93.3 (3)
O45—W5—O44—W2	−28.7 (9)	O57—W17—O58—W15	132.3 (10)
O43—W5—O45—W10	129.5 (8)	O58—W17—O39—W5	90.6 (3)
O44—W5—O45—W10	26.9 (8)	O39—W17—O6—W3	−57.8 (11)
O44—W5—O57—W17	11.7 (12)	O41—W17—O39—W5	−92.1 (3)
O40—W5—O57—W17	−71.1 (5)	O42—W17—O6—W3	125.7 (11)
O44—W5—O39—P1	−42.7 (5)	O6—W17—O58—W15	−23.9 (10)
O45—W5—O39—P1	44.0 (5)	O58—W17—O39—P1	−44.4 (5)
O40—W5—O44—W2	129.2 (9)	O6—W17—O57—W5	−14.9 (11)
O39—W5—O44—W2	55.8 (9)	O41—W17—O58—W15	53.6 (15)
O45—W5—O39—W17	−91.1 (3)	O6—W17—O41—W13	85.6 (5)
O43—W5—O57—W17	−172.7 (5)	O39—W17—O41—W13	2.3 (4)
O57—W5—O39—P1	133.6 (5)	O58—W17—O6—W3	26.0 (11)
O44—W5—O39—W17	−177.8 (3)	O42—W17—O57—W5	173.5 (5)
O40—W5—O39—P1	−133.8 (5)	O57—W17—O39—P1	−133.4 (5)
O40—W5—O39—W17	91.1 (3)	O41—W17—O39—P1	133.0 (5)
O39—W5—O57—W17	2.3 (4)	O39—W17—O58—W15	60.1 (10)
O57—W5—O39—W17	−1.6 (3)	O42—W17—O58—W15	−127.1 (10)
O18—W6—O33—W12	87.4 (5)	O39—W17—O57—W5	−2.3 (4)
O18—W6—O47—W14	−29.2 (9)	O41—W17—O57—W5	70.9 (5)
O33—W6—O47—W14	52.6 (14)	O57—W17—O6—W3	−45.8 (16)
O33—W6—O65—W12	−3.2 (3)	O6—W17—O39—P1	41.3 (5)
O18—W6—O65—W12	−95.7 (3)	O58—W17—O39—W13	−178.9 (3)
O47—W6—O65—P2	−39.6 (5)	O57—W17—O39—W13	92.1 (3)
O21—W6—O65—P2	−128.5 (5)	O58—W17—O41—W13	9.0 (11)
O18—W6—O65—P2	47.2 (5)	O57—W17—O41—W13	−69.3 (5)
O21—W6—O65—W12	88.7 (3)	O58—W17—O57—W5	−86.3 (5)
O65—W6—O47—W14	54.3 (8)	O42—W17—O41—W13	−170.2 (5)
O33—W6—O18—W9	−124.0 (9)	O15—W18—O13—W7	−72.7 (5)
O65—W6—O18—W9	−51.0 (8)	O53—W18—O35—W4	−128.9 (9)
O47—W6—O33—W12	6.5 (11)	O2—W18—O15—W9	−19.1 (11)
O21—W6—O33—W12	−77.1 (5)	O53—W18—O2—W1	130.2 (10)
O51—W6—O33—W12	−175.1 (5)	O13—W18—O15—W9	68.8 (5)
O33—W6—O65—P2	139.7 (5)	O34—W18—O2—W1	−54.1 (10)
O47—W6—O18—W9	33.8 (9)	O15—W18—O34—W9	3.1 (3)
O47—W6—O65—W12	177.6 (3)	O34—W18—O35—W4	56.0 (9)
O51—W6—O47—W14	−125.9 (8)	O35—W18—O2—W1	29.6 (10)
O21—W6—O47—W14	136.4 (9)	O34—W18—O15—W9	−4.7 (4)
O65—W6—O33—W12	4.7 (4)	O35—W18—O15—W9	−88.4 (5)
O51—W6—O18—W9	135.2 (9)	O35—W18—O34—W9	92.3 (3)
O34—W7—O13—W18	0.0 (4)	O13—W18—O35—W4	51.2 (14)
O14—W7—O13—W18	73.7 (5)	O15—W18—O35—W4	129.0 (9)
O13—W7—O34—W9	90.1 (3)	O15—W18—O34—P2	−132.5 (6)
O30—W7—O13—W18	−5.4 (11)	O35—W18—O34—P2	−43.3 (5)
O1—W7—O34—W18	91.0 (3)	O13—W18—O2—W1	−127.5 (10)
O34—W7—O30—W12	−56.4 (10)	O15—W18—O2—W1	−40.3 (16)
O13—W7—O34—P2	−135.5 (5)	O2—W18—O34—W9	177.3 (3)
O13—W7—O14—W9	−72.5 (5)	O2—W18—O34—P2	41.7 (5)
O30—W7—O34—W9	−92.0 (3)	O13—W18—O34—P2	134.7 (5)
O1—W7—O14—W9	4.1 (11)	O53—W18—O13—W7	−174.9 (5)
O66—W7—O30—W12	129.2 (10)	O2—W18—O35—W4	−28.5 (9)
O66—W7—O13—W18	174.2 (5)	O2—W18—O34—W7	−93.0 (3)
O30—W7—O34—W18	177.9 (3)	O13—W18—O34—W7	0.0 (3)
O34—W7—O14—W9	0.6 (4)	O15—W18—O34—W7	92.9 (3)
O1—W7—O13—W18	−83.0 (4)	O34—W18—O13—W7	0.0 (4)
O1—W7—O34—P2	−44.5 (5)	O35—W18—O13—W7	5.0 (10)
O30—W7—O14—W9	85.2 (5)	O2—W18—O13—W7	83.3 (4)
O1—W7—O34—W9	−178.9 (3)	O53—W18—O15—W9	170.5 (5)
O30—W7—O34—P2	42.4 (5)	O35—W18—O34—W7	−178.0 (3)
O14—W7—O1—W16	58.2 (12)	O13—W18—O34—W9	−89.8 (3)
O13—W7—O1—W16	134.0 (8)	O61—Co2—O62—C2	2.9 (9)
O14—W7—O34—W18	−90.4 (3)	O62—Co2—O61—C1	−0.8 (8)
O66—W7—O1—W16	−125.6 (8)	N1—Co2—O61—C1	175.6 (9)
O66—W7—O14—W9	−172.1 (5)	N2—Co2—O61—C1	89.8 (9)
O14—W7—O34—W9	−0.4 (3)	O62—Co2—N2—C3	−178.4 (8)
O14—W7—O34—P2	134.1 (5)	N1—Co2—N2—C3	6.6 (8)
O30—W7—O1—W16	−23.7 (8)	N3—Co2—N2—C3	−88.1 (9)
O14—W7—O30—W12	−129.4 (10)	O62—Co2—N3—C5	−101.3 (9)
O13—W7—O34—W18	0.0 (2)	N1—Co2—N3—C5	82.0 (9)
O1—W7—O30—W12	26.9 (10)	O61—Co2—N4—C6	158.7 (9)
O13—W7—O30—W12	−51.2 (16)	N3—Co2—N4—C6	−17.4 (9)
O34—W7—O1—W16	61.6 (8)	N1—Co2—N4—C6	−112.0 (9)
O5—W8—O27—P1	47.2 (5)	N4—Co2—N3—C5	−11.3 (9)
O7—W8—O27—W3	−2.3 (2)	N2—Co2—N3—C5	168.1 (9)
O5—W8—O27—W3	−93.7 (3)	N2—Co2—O62—C2	−86.8 (9)
O29—W8—O27—P1	−40.1 (5)	N3—Co2—O62—C2	179.5 (9)
O29—W8—O27—W3	179.0 (3)	N4—Co2—O62—C2	94.6 (9)
O27—W8—O5—W13	−56.8 (9)	N2—Co2—N1—C4	17.5 (9)
O7—W8—O27—P1	138.6 (5)	O61—Co2—N2—C3	95.8 (8)
O29—W8—O5—W13	26.9 (9)	N4—Co2—N1—C4	−164.0 (9)
O8—W8—O29—W11	−124.8 (9)	O62—Co2—N4—C6	72.9 (9)
O8—W8—O5—W13	129.7 (9)	N3—Co2—N1—C4	110.9 (9)
O7—W8—O5—W13	−130.6 (9)	O61—Co2—N1—C4	−72.3 (9)
O8—W8—O7—W3	−176.9 (5)	N4—Co2—O61—C1	−90.7 (9)
O3—W8—O27—W3	90.1 (3)	N10—Co1—O101—C14	175.4 (9)
O3—W8—O29—W11	137.9 (9)	O100—Co1—N7—C9	77.1 (8)
O27—W8—O29—W11	54.7 (9)	O101—Co1—N7—C9	162.7 (8)
O5—W8—O29—W11	−28.2 (9)	N8—Co1—N7—C9	−13.5 (8)
O5—W8—O7—W3	85.9 (5)	N10—Co1—N8—C10	79.1 (8)
O29—W8—O7—W3	6.7 (11)	N7—Co1—N10—C12	−162.6 (7)
O27—W8—O7—W3	3.4 (4)	O101—Co1—N10—C12	−69.7 (7)
O3—W8—O7—W3	−79.2 (5)	N7—Co1—N8—C10	−13.9 (8)
O3—W8—O27—P1	−129.1 (5)	N10—Co1—N7—C9	−108.6 (8)
O7—W8—O29—W11	51.6 (14)	N9—Co1—N8—C10	165.2 (8)
O15—W9—O18—W6	36.7 (15)	N8—Co1—N10—C12	111.6 (7)
O16—W9—O17—W14	129.6 (9)	N9—Co1—O101—C14	89.4 (9)
O34—W9—O17—W14	−55.5 (8)	O100—Co1—N9—C11	−179.9 (8)
O18—W9—O34—P2	−41.8 (5)	O101—Co1—N9—C11	94.5 (8)
O15—W9—O34—P2	132.5 (5)	N9—Co1—N10—C12	21.5 (7)
O16—W9—O14—W7	174.0 (5)	O100—Co1—N8—C10	−103.7 (8)
O18—W9—O17—W14	29.9 (9)	O101—Co1—O100—C13	2.6 (8)
O14—W9—O34—P2	−133.4 (5)	N8—Co1—N9—C11	−89.3 (8)
O15—W9—O17—W14	−128.4 (9)	N10—Co1—N9—C11	6.1 (8)
O17—W9—O34—P2	43.0 (5)	N7—Co1—O101—C14	−91.3 (9)
O15—W9—O14—W7	71.8 (5)	N7—Co1—O100—C13	95.6 (8)
O15—W9—O34—W18	−3.1 (3)	N9—Co1—O100—C13	−88.5 (8)
O17—W9—O34—W18	−92.6 (3)	O100—Co1—O101—C14	−1.6 (8)
O16—W9—O18—W6	−133.0 (9)	N8—Co1—O100—C13	−178.9 (8)
O18—W9—O34—W18	−177.5 (3)	O39—P1—O27—W8	−62.9 (5)
O14—W9—O17—W14	−46.6 (14)	O39—P1—O26—W10	65.1 (6)
O17—W9—O18—W6	−33.7 (9)	O28—P1—O27—W8	53.8 (5)
O18—W9—O14—W7	−85.2 (5)	O28—P1—O26—W10	−52.0 (6)
O14—W9—O34—W7	0.4 (3)	O39—P1—O27—W3	64.8 (5)
O18—W9—O34—W7	91.9 (3)	O27—P1—O26—W15	53.9 (5)
O17—W9—O15—W18	88.8 (5)	O27—P1—O26—W10	−177.5 (4)
O34—W9—O18—W6	50.5 (8)	O27—P1—O28—W11	−52.8 (5)
O34—W9—O14—W7	−0.6 (4)	O26—P1—O27—W8	179.9 (4)
O14—W9—O18—W6	123.7 (9)	O26—P1—O28—W11	−178.7 (4)
O15—W9—O34—W7	−93.7 (3)	O27—P1—O39—W5	179.1 (4)
O14—W9—O34—W18	91.0 (3)	O27—P1—O28—W2	178.2 (4)
O17—W9—O34—W7	176.7 (3)	O39—P1—O28—W2	−64.8 (5)
O17—W9—O14—W7	−9.8 (12)	O26—P1—O39—W13	179.8 (4)
O34—W9—O15—W18	4.7 (4)	O27—P1—O39—W13	59.5 (5)
O18—W9—O15—W18	19.1 (12)	O39—P1—O28—W11	64.1 (5)
O16—W9—O15—W18	−171.3 (5)	O26—P1—O28—W2	52.3 (5)
O14—W9—O15—W18	−68.2 (5)	O26—P1—O39—W5	−60.6 (5)
O45—W10—O23—W2	31.9 (9)	O39—P1—O26—W15	−63.5 (5)
O54—W10—O45—W5	−128.7 (8)	O26—P1—O27—W3	−52.5 (5)
O21—W10—O23—W2	−132.8 (9)	O28—P1—O27—W3	−178.5 (4)
O26—W10—O45—W5	58.4 (8)	O28—P1—O26—W15	179.4 (4)
O26—W10—O23—W2	−50.0 (9)	O28—P1—O39—W13	−59.8 (6)
O21—W10—O25—W15	79.0 (4)	O26—P1—O39—W17	60.2 (6)
O21—W10—O26—P1	127.3 (5)	O28—P1—O39—W17	−179.4 (4)
O25—W10—O23—W2	−42.4 (13)	O27—P1—O39—W17	−60.1 (5)
O23—W10—O45—W5	−27.0 (8)	O28—P1—O39—W5	59.8 (5)
O25—W10—O45—W5	132.0 (8)	O65—P2—O34—W18	−179.4 (4)
O45—W10—O26—P1	−48.6 (5)	O65—P2—O34—W9	59.9 (5)
O54—W10—O23—W2	127.4 (9)	O50—P2—O34—W18	−59.4 (6)
O25—W10—O26—P1	−139.7 (5)	O36—P2—O34—W18	60.6 (5)
O23—W10—O26—P1	37.3 (5)	O36—P2—O65—W6	52.2 (5)
O23—W10—O26—W15	179.3 (2)	O65—P2—O50—W1	−178.7 (4)
O25—W10—O26—W15	2.2 (3)	O34—P2—O65—W6	−64.3 (5)
O45—W10—O26—W15	93.4 (2)	O50—P2—O34—W9	179.9 (4)
O45—W10—O25—W15	−84.8 (4)	O50—P2—O65—W12	−51.9 (5)
O23—W10—O25—W15	−11.1 (11)	O34—P2—O50—W16	−64.8 (6)
O54—W10—O25—W15	179.0 (4)	O36—P2—O50—W16	178.9 (4)
O21—W10—O26—W15	−90.8 (3)	O65—P2—O50—W16	52.3 (5)
O26—W10—O25—W15	−3.2 (4)	O50—P2—O65—W6	178.8 (4)
O55—W11—O29—W8	27.3 (9)	O36—P2—O50—W1	−52.0 (5)
O46—W11—O29—W8	−137.0 (9)	O36—P2—O65—W12	−178.5 (4)
O24—W11—O29—W8	−49.1 (14)	O36—P2—O34—W7	−179.0 (4)
O60—W11—O29—W8	123.9 (9)	O50—P2—O34—W7	61.1 (6)
O60—W11—O55—W13	−128.5 (11)	O36—P2—O34—W9	−60.2 (5)
O28—W11—O29—W8	−54.6 (9)	O50—P2—O36—W4	52.0 (5)
O29—W11—O28—P1	38.8 (5)	O65—P2—O36—W4	178.5 (4)
O29—W11—O24—W2	−10.5 (11)	O34—P2—O36—W14	64.8 (5)
O55—W11—O24—W2	−86.1 (5)	O50—P2—O36—W14	−178.6 (4)
O28—W11—O24—W2	−4.8 (4)	O65—P2—O36—W14	−52.1 (5)
O24—W11—O28—W2	3.3 (3)	O34—P2—O65—W12	65.0 (5)
O24—W11—O55—W13	131.8 (11)	O34—P2—O50—W1	64.3 (5)
O29—W11—O28—W2	−178.9 (3)	O65—P2—O34—W7	−58.9 (5)
O46—W11—O28—W2	−89.4 (3)	O34—P2—O36—W4	−64.6 (5)
O46—W11—O28—P1	128.4 (5)	Co2—O61—C1—O64	−178.3 (11)
O29—W11—O55—W13	−26.3 (11)	Co2—O61—C1—C2	−1.0 (13)
O55—W11—O28—P1	−47.1 (5)	Co2—O62—C2—C1	−4.0 (13)
O60—W11—O24—W2	176.5 (4)	Co2—O62—C2—O63	175.1 (11)
O46—W11—O24—W2	77.0 (5)	Co1—O100—C13—O103	175.6 (10)
O24—W11—O28—P1	−139.0 (5)	Co1—O100—C13—C14	−2.9 (12)
O28—W11—O55—W13	58.1 (11)	Co1—O101—C14—O102	−177.1 (11)
O55—W11—O28—W2	95.1 (3)	Co1—O101—C14—C13	0.5 (13)
O48—W12—O65—P2	37.4 (5)	Co2—N1—C4—C3	−37.8 (13)
O30—W12—O33—W6	−85.9 (5)	Co2—N2—C3—C4	−29.1 (13)
O48—W12—O33—W6	−13.4 (11)	Co2—N3—C5—C6	36.7 (12)
O33—W12—O65—W6	3.1 (3)	Co2—N4—C6—C5	42.1 (13)
O32—W12—O33—W6	177.7 (5)	Co1—N7—C9—C10	37.0 (12)
O65—W12—O33—W6	−4.7 (4)	Co1—N8—C10—C9	38.1 (11)
O32—W12—O48—W16	127.9 (10)	Co1—N9—C11—C12	−32.5 (12)
O30—W12—O65—P2	−48.4 (5)	Co1—N10—C12—C11	−45.3 (11)
O31—W12—O65—W6	−89.4 (3)	O61—C1—C2—O62	3.4 (16)
O33—W12—O65—P2	−139.3 (5)	O64—C1—C2—O62	−179.3 (12)
O30—W12—O65—W6	93.9 (3)	O64—C1—C2—O63	2 (2)
O65—W12—O30—W7	56.7 (10)	O61—C1—C2—O63	−175.8 (12)
O31—W12—O33—W6	78.5 (5)	N2—C3—C4—N1	43.2 (15)
O65—W12—O48—W16	−49.3 (10)	N3—C5—C6—N4	−50.8 (14)
O33—W12—O30—W7	129.8 (10)	N7—C9—C10—N8	−48.6 (13)
O48—W12—O65—W6	179.8 (3)	N9—C11—C12—N10	50.9 (13)
O31—W12—O48—W16	−132.9 (10)	O100—C13—C14—O101	1.7 (16)
O30—W12—O48—W16	32.2 (10)	O103—C13—C14—O102	0.9 (19)
O33—W12—O48—W16	−40.9 (16)	O100—C13—C14—O102	179.4 (11)
O32—W12—O30—W7	−131.2 (10)	O103—C13—C14—O101	−176.9 (12)
O48—W12—O30—W7	−28.6 (10)	N5—C7—C7^i^—N5^i^	180.0 (10)
O31—W12—O65—P2	128.2 (5)		

Symmetry code: (i) −*x*+2, −*y*+1, −*z*+2.

### Hydrogen-bond geometry (Å, º)

**Table:**

*D*—H···*A*	*D*—H	H···*A*	*D*···*A*	*D*—H···*A*
N2—H2*A*···O100^ii^	0.90	2.59	3.314 (14)	138
N2—H2*A*···O103^ii^	0.90	2.07	2.940 (14)	163
N2—H2*B*···O38	0.90	2.26	3.115 (13)	157
O2*W*—H2*WA*···O57^iii^	0.85	2.44	2.919 (17)	117
N3—H3*C*···O103^ii^	0.90	2.48	2.994 (14)	117
O2*W*—H2*WB*···O6*W*	0.85	2.04	2.86 (3)	162
N4—H4*D*···O1*W*	0.90	2.06	2.939 (18)	167
O3*W*—H3*WA*···O102^ii^	0.85	2.16	3.000 (18)	172
O4*W*—H4*WA*···O12^iv^	0.85	2.14	2.983 (18)	173
N7—H7*D*···O5*W*	0.90	2.11	3.010 (17)	176
O4*W*—H4*WB*···O42	0.85	2.05	2.891 (16)	173
N5—H8*A*···O102	0.89	1.86	2.740 (15)	170
N5—H8*B*···O45^iii^	0.89	2.23	3.076 (13)	158
N5—H8*B*···O57^iii^	0.89	2.54	3.166 (14)	128
N5—H8*C*···O2*W*	0.89	2.05	2.881 (17)	155
N8—H8*E*···O7*W*	0.90	2.23	3.07 (2)	154
N9—H9*C*···O63^ii^	0.90	2.12	3.023 (13)	175
N9—H9*D*···O49^iii^	0.90	2.20	3.088 (13)	170
C3—H3*A*···O29	0.97	2.49	3.417 (17)	160
C4—H4*B*···O10	0.97	2.47	3.396 (18)	160
C11—H11*A*···O33^iii^	0.97	2.52	3.376 (17)	147
C12—H12*A*···O25^iii^	0.97	2.51	3.297 (16)	139

Symmetry codes: (ii) −*x*+1, −*y*+2, −*z*+1; (iii) −*x*+1, −*y*+1, −*z*+1; (iv) −*x*, −*y*+1, −*z*+1.
